# FUNDC1 protects against doxorubicin-induced cardiomyocyte PANoptosis through stabilizing mtDNA via interaction with TUFM

**DOI:** 10.1038/s41419-022-05460-x

**Published:** 2022-12-05

**Authors:** Yaguang Bi, Haixia Xu, Xiang Wang, Hong Zhu, Junbo Ge, Jun Ren, Yingmei Zhang

**Affiliations:** 1 Department of Cardiology, Zhongshan Hospital, Fudan University, Shanghai Institute of Cardiovascular Diseases, 200032 Shanghai, China; 2 National Clinical Research Center for Interventional Medicine, 200032 Shanghai, China; 3 Department of Cardiology, Affiliated Hospital of Nantong University, 226001 Nantong, Jiangsu China; 4 Laboratory of Oral Microbiota and Systemic Diseases, Shanghai Ninth People’s Hospital, College of Stomatology, Shanghai Jiao Tong University School of Medicine, 200125 Shanghai, China; 5 Department of Laboratory Medicine and Pathology, University of Washington, Seattle, WA 98195 USA

**Keywords:** Cardiomyopathies, Cardiovascular diseases

## Abstract

Doxorubicin (DOX) is an effective anthracycline chemotherapeutic anticancer drug with its life-threatening cardiotoxicity severely limiting its clinical application. Mitochondrial damage-induced cardiomyocyte death is considered an essential cue for DOX cardiotoxicity. FUN14 domain containing 1 (FUNDC1) is a mitochondrial membrane protein participating in the regulation of mitochondrial integrity in multiple diseases although its role in DOX cardiomyopathy remains elusive. Here, we examined whether PANoptosis, a novel type of programmed cell death closely associated with mitochondrial damage, was involved in DOX-induced heart injury, and FUNDC1-mediated regulation of cardiomyocyte PANoptosis, if any. FUNDC1 was downregulated in heart tissues in patients with dilated cardiomyopathy (DCM) and DOX-challenged mice. FUNDC1 deficiency aggravated DOX-induced cardiac dysfunction, mitochondrial injury, and cardiomyocyte PANoptosis. Further examination revealed that FUNDC1 countered cytoplasmic release of mitochondrial DNA (mtDNA) and activation of PANoptosome through interaction with mitochondrial Tu translation elongation factor (TUFM), a key factor in the translational expression and repair of mitochondrial DNA, via its 96–133 amino acid domain. TUFM intervention reversed FUNDC1-elicited protection against DOX-induced mtDNA cytosolic release and cardiomyocyte PANoptosis. Our findings shed light toward a beneficial role of FUNDC1 in DOX cardiotoxicity and cardiomyocyte PANoptosis, thus offering therapeutic promises in DOX-induced cardiotoxicity.

## Introduction

Doxorubicin (DOX) is a widely employed chemotherapeutic agent in a variety of cancers, including leukemias and solid tumors [1, 2]. Unfortunately, DOX treatment also causes adverse drug reactions with cardiovascular toxicity being one of the most common and life-threatening-side effect [3–6]. DOX cardiotoxicity leads to dilated cardiomyopathy (DCM) and heart failure (HF), ultimately limiting its clinical utilization [7, 8]. Although the underlying mechanisms behind DOX-evoked cardiotoxicity have been intensively studied [9, 10], feasible and effective targets for preventing or reversing DOX cardiotoxicity are still lacking.

Among various contemporary theories postulated for DOX cardiotoxicity, mitochondrial perturbation appears to receive predominant attention [11]. Upon DOX challenge, mitochondria represent the most severely impaired intracellular organelles, with mitochondrial injury being one of the first events for DOX cardiotoxicity [12]. Mitochondrial dysfunction along with mitochondrial DNA (mtDNA) damage provokes multiple forms of programmed cell death (PCD) in cardiomyocytes under DOX insult, including apoptosis [13, 14], ferroptosis [15, 16], and necroptosis [17, 18]. The mammalian mitochondrial membrane protein FUN14 domain containing 1 (FUNDC1) is a mitophagy receptor which interacts with and recruits LC3 to mitochondria for the induction of mitophagy [19]. Several studies have shown that FUNDC1 also exerts vital roles in a wide array of biological events including regulation of mitochondrial dynamics [20], maintenance of mitochondrial calcium homeostasis [21], and cellular plasticity through preservation of oxidative bioenergetics, governance of ROS production and cell proliferation [22], ultimately alleviating mitochondrial damage in multiple diseases [21, 23, 24]. Nevertheless, the role of FUNDC1 in DOX-induced cardiotoxicity has not been clarified.

PANoptosis is not only a newly recognized form of proinflammatory programmed cell death, but also an emerging concept highlighting the crosstalk and coordination among three cell death patterns namely, pyroptosis, apoptosis, and necroptosis [25, 26]. PANoptosis is driven through a multiprotein complex termed PANoptosome to enable crosstalk and regulation among these processes [27, 28]. Recent studies have shown that the PANoptosome contains molecules critical for pyroptosis, apoptosis, and necroptosis including Gasdermin D (GSDMD), Caspase1, Caspase3, Caspase8, RIP kinase 1 (RIPK1), and RIP kinase 3 (RIPK3), which enables activation of all three pathways to execute cell death [29, 30]. It is believed that the conceptualized complex would form a flexible skeleton, whereby the core components of different cell death patterns may be recruited to execute cell death [27, 29]. Mechanistic approach has later revealed several molecules capable of regulating pyroptosis, apoptosis, and necroptosis. In particular, Z-DNA-binding protein 1 (ZBP1) is crucial for activation of all three pathways [25, 29]. Moreover, the cytosolic double-stranded DNA (dsDNA) senser AIM2 was found to be crucial for activation of PANoptosis through formation of AIM2-PANoptosome [31, 32]. AIM2, inflammasome sensor pyrin, and ZBP1 are members of the large multiprotein complex PANoptosome, along with Caspase1, Caspase8, RIPK3, and RIPK1 [31, 32]. AIM2 was also demonstrated to be turned on by mtDNA release in metabolic diseases [33] in addition to its role in sensing dsDNA of viral invasion. However, whether the new form of programmed cell death PANoptosis participates in cardiovascular pathology especially DOX cardiotoxicity remains elusive.

To this end, this study was designed to evaluate the impact of FUNDC1 ablation on DOX cardiotoxicity and the mechanism involved with a focus on PANoptosis in cardiomyocytes. Our data revealed the downregulation of FUNDC1 in heart tissues from patients with dilated cardiomyopathy (DCM) and mice with DOX challenge. FUNDC1 deficiency aggravated DOX-induced cardiac dysfunction, mitochondrial damage and cardiomyocyte PANoptosis. In an effort to discern the mechanistic basis behind FUNDC1-offered regulation of mitochondrial damage during DOX challenge, possible contribution of mtDNA release to activate PANoptosome was examined. We found that FUNDC1 was able to interact with mitochondrial Tu translation elongation factor (TUFM), a key factor in the translational expression and repair of mitochondrial DNA [34, 35] to fend off mtDNA cytosolic release and cardiomyocyte PANoptosis evoked by DOX. These findings support a protective role of FUNDC1 in DOX-driven cardiotoxicity and cardiomyocyte PANoptosis.

## Results

### FUNDC1 deficiency aggravated DOX-induced cardiac dysfunction in mice

**Fig. 1:**
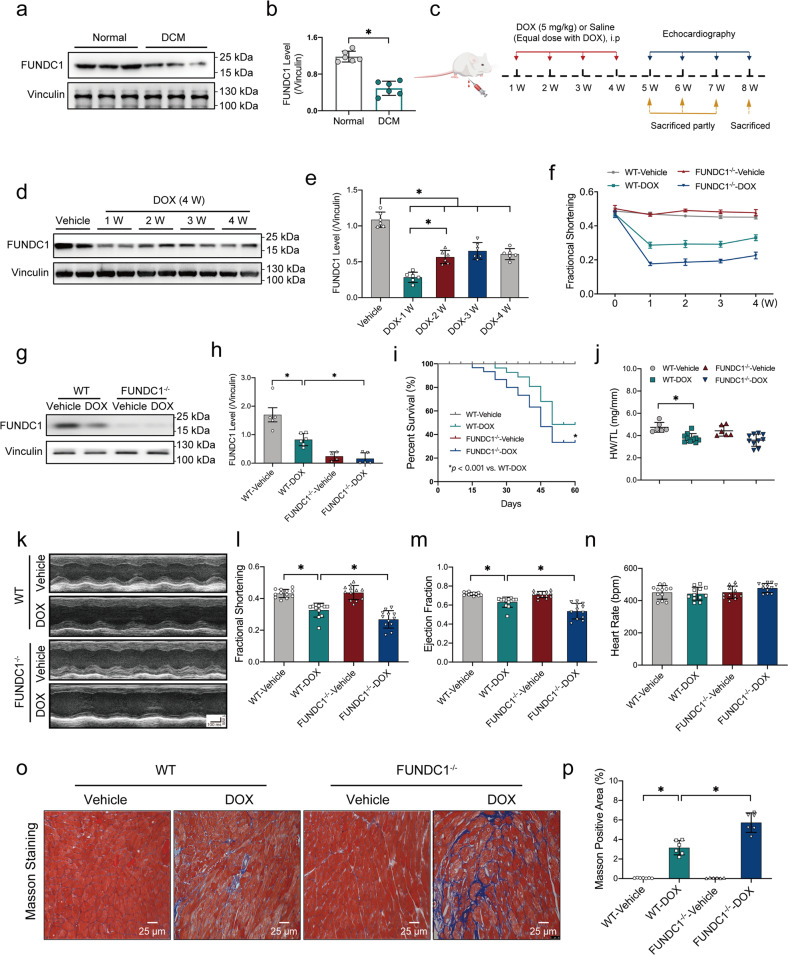
FUNDC1 deficiency aggravated DOX-induced cardiac dysfunction in mice. **a**, **b** Representative immunoblot and quantitative histogram of FUNDC1 levels in cardiac tissues from patients with dilated cardiomyopathy (DCM) or healthy donors (Vinculin as the loading control) (*n* = 6/group). **c** Scheme of DOX challenge in mice and time points of functional assessments. **d**, **e** Representative immunoblot and quantitative histogram of FUNDC1 in cardiac tissues of mice at 1, 2, 3, and 4 W following completion of DOX challenge (4 weeks) (Vinculin as a loading control) (*n* = 6/group). **f** Echocardiographic analysis of fractional shortening in mice at 1, 2, 3, and 4 W following completion of DOX challenge (*n* = 6 and 11 for vehicle-treated and DOX groups, respectively). **g**, **h** Representative immunoblot and quantitative histogram of FUNDC1 in heart tissues of mice at 1 W following completion of DOX challenge (Vinculin as a loading control) (*n* = 6/group). **i** Survival rate (*n* = 20/group). **j** Ratio of heart weight (HW) to tibial length (TL) (*n* = 6 and 11 for vehicle-treated and DOX groups, respectively). **k** Representative echocardiographic images of cardiac function, scale bar = 100 ms/1 mm. **l**–**n** Quantitative analysis of fraction shortening, ejection fraction and heart rate (*n* = 12/group). **o**, **p** Representative images and quantitative analysis of Masson Trichrome staining (*n* = 6/group), scale bar = 25 μm. Mean ± SEM, *P < 0.05 between indicated groups, Student’s *t* test was used in (**b**), log-rank test was used in (**i**), and one-way ANOVA followed by a Tukey’s test was used in (**e**, **h**, **j**, **i**–**n**, **p**).

To discern levels of FUNDC1 in DCM and DOX-challenged mouse hearts, heart tissues from DCM patients and healthy donors were evaluated. Levels of FUNDC1 were significantly downregulated in heart samples from DCM patients compared with healthy donors (Fig. 1a, b). Next, a mouse model of DOX-induced cardiac injury was established using intraperitoneal injection with DOX (5 mg/kg, four doses, once per week) or an equal dose of saline prior to the assessment of myocardial function at 1, 2, 3 and 4 W following completion of DOX challenge (for 4 weeks) (scheme shown in Fig. 1c). Downregulation of FUNDC1 was observed in heart tissues at 1, 2, 3 and 4 W following DOX exposure, with a more pronounced response at 1 W (Fig. 1d, e). Moreover, echocardiographic analysis revealed that DOX challenge impaired fractional shortening at 1, 2, 3 and 4 W following DOX exposure, with the most pronounced responsiveness at 1 W (Fig. 1f). Thus, 1 week following completion of the 4-week DOX treatment was chosen for further analysis (with the exception of Masson staining for fibrosis analysis). Besides, AC-16 cardiomyocytes were treated with DOX (1 μM, 24 h) to evaluate the expression of FUNDC1. DOX challenge downregulated FUNDC1 in AC-16 cardiomyocytes (Supplementary Fig. S1a, b). These results denoted the downregulation of FUNDC1 in human DCM heart tissues, DOX-challenged mouse heart tissues and human cardiomyocytes.

To evaluate the possible role of FUNDC1 in DOX cardiotoxicity, FUNDC1 knockout (FUNDC1^−/−^) and wild-type (WT) mice were challenged with DOX. FUNDC1 knockout efficiency was verified using western blot analysis (Fig. 1g, h). DOX challenge overtly dropped mouse survival, with a more pronounced response in FUNDC1 ablation (especially in the initiation of mortality), although FUNDC1 knockout itself did not affect mouse survival in the absence of DOX challenge (Fig. 1i). Meanwhile, DOX challenge overtly decreased heart weight-to-tibial length ratio (HW/TL), with FUNDC1 ablation has little effect on HW/TL (Fig. 1j). In addition, echocardiographic analysis exhibited that DOX insult decreased fractional shortening, and ejection fraction (Fig. 1k–m) (consistent with Fig. 1f), in conjunction with an elevated left ventricular end-diastolic diameter (LVEDD), and left ventricular end-systolic dimension (LVESD) (Supplementary Fig. S1c, d). Although FUNDC1 ablation itself failed to affect echocardiographic indices, it aggravated DOX-induced changes in cardiac geometry and function (Fig. 1k–m and Supplementary Fig. S1c, d). Neither DOX challenge nor FUNDC1 removal (or both) overtly affected heart rate and left ventricular posterior wall diameter in diastole (LVPWD) in all groups (Fig. 1n and Supplementary Fig. S1e). Moreover, Masson staining (performed at 4 W following the completion of DOX challenge) revealed an overtly increased fibrosis area in response to DOX insult, the effect of which was exacerbated by FUNDC1 ablation with little effect from FUNDC1 deficiency itself (Fig. 1o, p). These results suggested that FUNDC1 deletion aggravated DOX-induced cardiac injury and cardiac dysfunction in mice.

### FUNDC1 deficiency exacerbated DOX-induced cardiomyocyte contractile dysfunction in mice

**Fig. 2:**
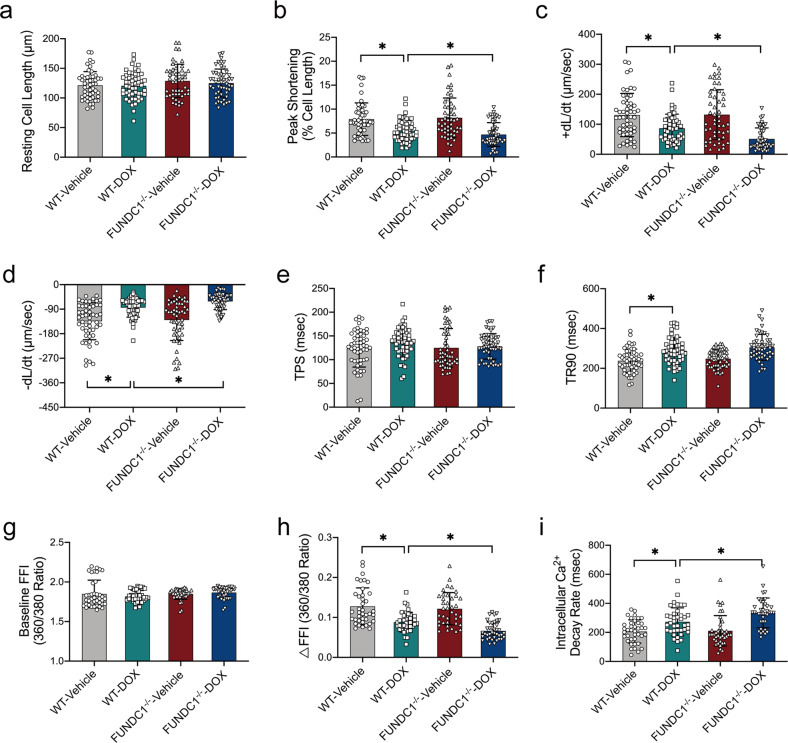
FUNDC1 deficiency exacerbated DOX-induced cardiomyocyte contractile dysfunction in mice. Adult cardiomyocytes (AMCMs) isolated from WT or FUNDC1^−/−^ mice with or without DOX insult, prior to mechanical assessment. **a** Resting cell length. **b** Peak shortening (PS). **c** Maximal velocity of shortening (+dL/dt). **d** Maximal velocity of re-lengthening (−dL/dt). **e** Time-to-peak shortening (TPS). **f** Time-to-90% re-lengthening (TR_90_). **g** Baseline Fura-2 fluorescence intensity (FFI). **h** Electrically stimulated rise in FFI (ΔFFI) and **i** Intracellular Ca^2+^ decay rate. Mean ± SEM, *n* = 50–60/group, *P < 0.05 between indicated groups, one-way ANOVA followed by a Tukey’s test was used in (**a**–**i**).

Consistent with the echocardiographic findings, adult cardiomyocytes (AMCMs) isolated from DOX-insulted mice exhibited overtly dampened contractile function, as manifested by decreased peak shortening (PS), maximal velocity of shortening/re-lengthening (± dL/dt), and prolonged time-to-90% re-lengthening (TR_90_), the effect of which was exacerbated by FUNDC1 ablation (FUNDC1 ablation had a little notable effect on TR_90_), with little effect from FUNDC1 removal itself. Resting cell length and time-to-PS (TPS) remained unaffected in all experimental groups (Fig. 2a–f).

Next, intracellular Ca^2+^ handling was evaluated using Fura-2 fluorescence microscopy. Adult cardiomyocytes from DOX-challenged mice displayed overtly decreased intracellular Ca^2+^ release in response to electrical stimuli (ΔFFI) and prolonged intracellular Ca^2+^ decay, the effect of which was aggravated by FUNDC1 ablation with little effect from FUNDC1 deficiency itself. Resting intracellular Ca^2+^ (baseline FFI) remained unchanged in all groups (Fig. 2g–i). Levels of FUNDC1 were validated in isolated AMCMs from WT and FUNDC1^−/−^ mice challenged with DOX using western blot analysis. The results indicated the downregulation of FUNDC1 levels following DOX challenge, the effect of which was masked by FUNDC1 ablation (Supplementary Fig. S1f, g). These results indicated that FUNDC1 deficiency exacerbated DOX-induced cardiomyocyte contractile dysfunction.

### FUNDC1 deficiency worsened DOX-induced cardiomyocyte mitochondrial injury in vivo and in vitro

**Fig. 3:**
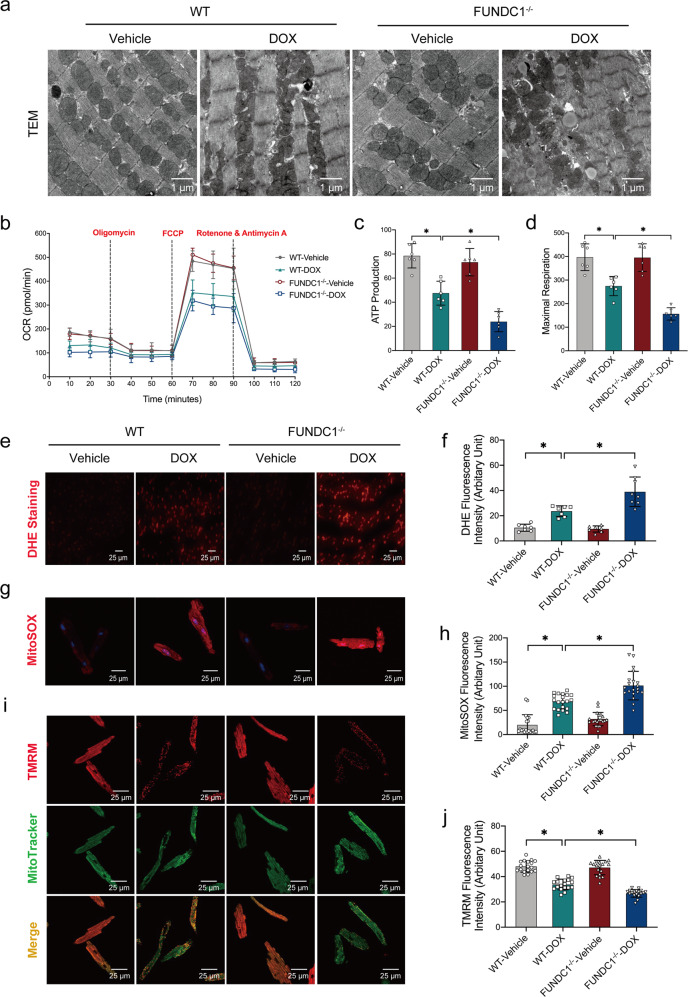
FUNDC1 deficiency worsened DOX-induced cardiomyocyte mitochondrial injury in vivo and in vitro. **a** Representative TEM images of murine heart tissues, scale bar = 1 μm. **b**–**d** Mitochondrial OCR and statistical analysis of ATP production and maximal respiration (*n* = 6/group), scale bar = 25 μm. **e**, **f** Representative images and quantitative analysis of DHE staining (*n* = 6–7/group), scale bar = 25 μm. **g**, **h** Representative images and quantitative analysis of MitoSOX staining (*n* = 20/group), scale bar = 25 μm. **i**, **j** Representative images and quantitative analysis of TMRM staining (*n* = 20/group), scale bar = 25 μm. Mean ± SEM, *P < 0.05 between indicated groups, one-way ANOVA followed by a Tukey’s test was used in (**c**, **d**, **f**, **h**, **j**).

To evaluate the effect of DOX on cardiomyocyte mitochondrial function, transmission electron microscopy (TEM) was performed. Our data revealed pronounced myocardial damage, including disrupted mitochondria, distortion of sarcomeres and myofilaments following DOX challenge, with or without FUNDC1 deletion (Fig. 3a). Moreover, DOX challenge impaired O_2_ consumption rate (OCR) in AMCMs (from DOX-treated mice) including ATP production and maximal respiration, the effects of which were accentuated by FUNDC1 ablation with little effect from FUNDC1 deficiency itself (Fig. 3b–d). Next, cytosolic ROS level was evaluated using DHE staining in vivo, while mitochondrial ROS production and mitochondrial membrane potential (MMP) were examined using the fluorescent dyes MitoSOX and TMRM, respectively, in AMCMs isolated from WT and FUNDC1 deficient mice. DOX challenge overtly elevated ROS levels in heart tissues (Fig. 3e, f), promoted mitochondrial ROS production (Fig. 3g, h), along with collapsed MMP in AMCMs (Fig. 3i, j), the effects of which were aggravated by FUNDC1 ablation with little effect from FUNDC1 deficiency itself (Fig. 3e–j). These findings favored that FUNDC1 deficiency worsened DOX-induced cardiomyocyte mitochondrial injury in vivo and in vitro.

### FUNDC1 protected against DOX-induced cardiomyocyte mtDNA release, mitochondrial damage, and cardiomyocyte death

**Fig. 4:**
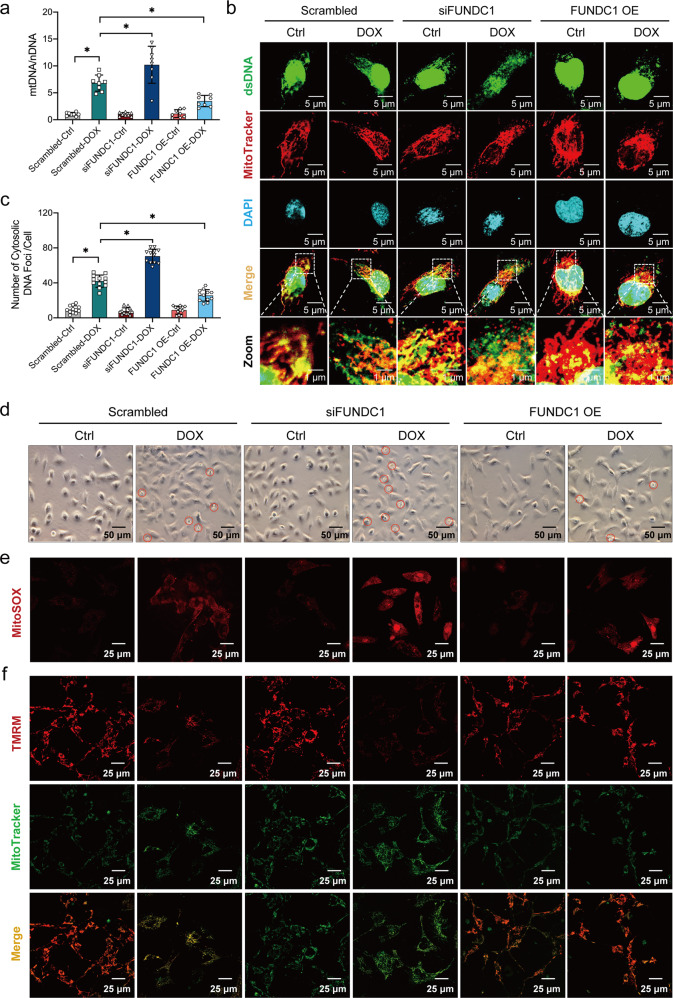
FUNDC1 protected against DOX-induced cardiomyocyte mtDNA release, mitochondrial damage, and cardiomyocyte death. **a** Ratio of mtDNA-to-nDNA in AC-16 cardiomyocytes with FUNDC1 deletion or overexpression in the presence or absence of DOX challenge (*n* = 8/group). **b**, **c** Immunofluorescence staining images and quantitative analysis of mtDNA released to the cytoplasm (portion of dsDNA not co-localized with MitoTracker or DAPI) (*n* = 12/group), scale bar = 5 μm, scale bar in zoom = 1 μm. **d** Cell state observation (red circles denoting dead cells), scale bar = 50 μm. **e** Representative images of MitoSOX staining (*n* = 20/group), scale bar = 25 μm. **f** Representative images of TMRM staining (*n* = 20/group), scale bar = 25 μm. Mean ± SEM, *P < 0.05 between groups, one-way ANOVA followed by a Tukey’s test was used in (**a**–**c**).

DOX is known to trigger mtDNA release into the cytoplasm [5]. To evaluate mtDNA release following DOX insult and FUNDC1 deficiency or overexpression, cytosolic and nuclear fractions were used to quantitate level of DNA containing specific mitochondrial (mtCOI) and nuclear (18 S rDNA) genes using qPCR (primer sequence listed in Supplementary Table S1). AC-16 cells were silenced or transfected with FUNDC1 prior to DOX exposure (Supplementary Fig. S2a, b). DOX exposure increased the cytosolic mtDNA-to-nDNA ratio in AC-16 cardiomyocytes, suggesting provoked mtDNA release to the cytoplasm, the effect of which was more pronounced by siRNA-mediated FUNDC1 knockdown, while such response was mitigated by FUNDC1 overexpression (Fig. 4a). Along the same line, immunofluorescence staining revealed that DOX challenge boosted the level of dsDNA not co-localized with MitoTracker or DAPI (denoting mtDNA release into the cytoplasm), the effect of which was deteriorated and mitigated by FUNDC1 ablation and overexpression, respectively (Fig. 4b, c). Moreover, DOX insult evoked cell death (shrunken and brightened cells or the PI-positive cell), the effect of which was worsened and alleviated by FUNDC1 ablation and overexpression, respectively (Fig. 4d and Supplementary Fig. S2c, d). MitoSOX and TMRM staining revealed that FUNDC1 ablation aggravated DOX-induced mitochondrial ROS production and MMP collapse, while FUNDC1 overexpression exhibited the opposite effect (Fig. 4e, f and Supplementary Fig. S2e, f). These data illustrated that FUNDC1 protected against DOX-elicited mtDNA release and mitochondrial damage in cardiomyocytes.

### FUNDC1 deficiency promoted DOX-induced cardiomyocyte apoptosis, necroptosis, and pyroptosis (PANoptosis)

**Fig. 5:**
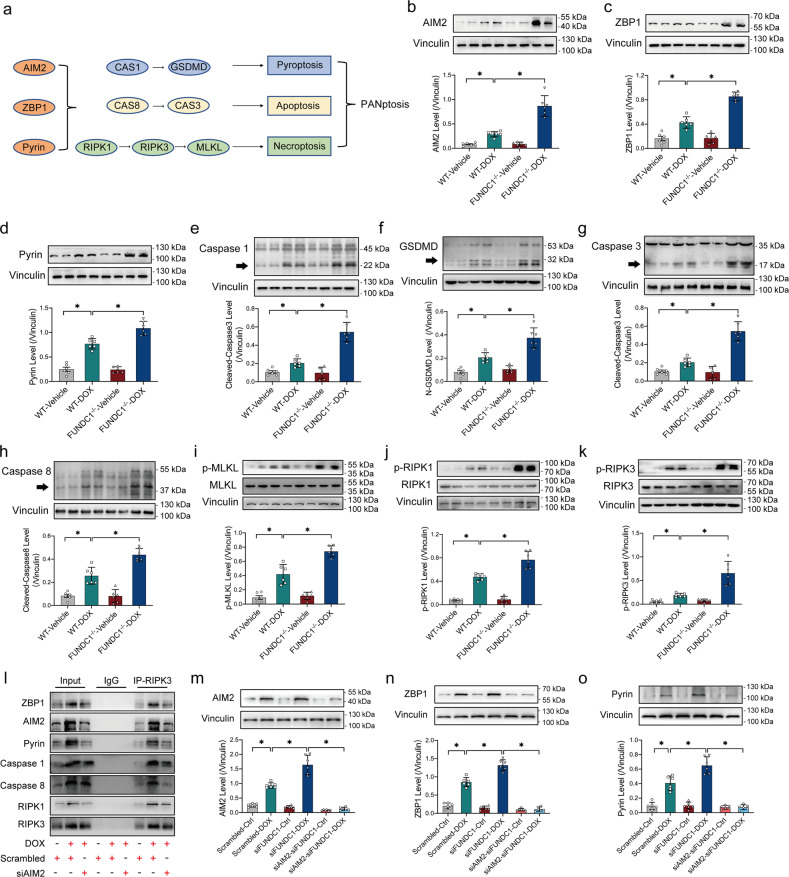
FUNDC1 deficiency promoted DOX-induced cardiomyocyte apoptosis, necroptosis, and pyroptosis (PANoptosis). **a** Scheme of PANoptosome (AIM2, ZBP1, Pyrin)-induced pyroptosis, apoptosis and necroptosis (PANoptosis). **b**–**d** Representative immunoblots and quantitative histograms of AIM2, ZBP1, Pyrin (members of PANoptosome) (Vinculin as the loading control). **e**, **f** Representative immunoblots and quantitative histograms of pyroptosis markers Caspase1 and GSDMD (Vinculin as the loading control). **g**, **h** Representative immunoblots and quantitative histograms of apoptosis markers Caspase8 and Caspase3 (Vinculin as the loading control). **i**–**k** Representative immunoblots and quantitative histograms of total and phosphorylation forms of MLKL, RIPK1, RIPK3 (necroptosis markers) (Vinculin as the loading control). **l** Co-IP analysis of ZBP1, AIM2, Pyrin, Caspase1, Caspase8, RIPK1, and RIPK3 in RIPK3 immune complexes in DOX-treated AC-16 cardiomyocytes with AIM2 siRNA or scrambled siRNA transfection. **m**–**o** Representative immunoblots and quantitative histograms of AIM2, ZBP1, Pyrin in AC-16 cardiomyocytes exposed to DOX, under FUNDC1 deficiency together with or without AIM2 ablation (Vinculin as the loading control). Mean ± SEM, *n* = 6 per group, *P < 0.05 between indicated groups. One-way ANOVA followed by a Tukey’s test was used in (**b**–**k**, **m**–**o**).

PANoptosis is a newly recognized programmed cell death pattern [25, 26], although its role in DOX cardiotoxicity remains unknown. PANoptosis is driven through a multiprotein complex activated by the DNA sensers AIM2, ZBP1, and Pyrin to form a flexible skeleton to recruit Caspase1, Caspase3, Caspase8, RIPK1, and RIPK3, ultimately resulting in the execution of concurrent pyroptosis, apoptosis and necroptosis (Fig. 5a). Hence, PANoptosis was evaluated in DCM patient heart tissues. Our result indicated activation of PANoptosis in heart tissues of DCM patients manifested as increased levels of AIM2, ZBP1, Pyrin (essential members of PANoptosome) (Supplementary Fig. S3a–c), active forms of Caspase1 and GSDMD (pyroptosis markers) (Supplementary Fig. S3d, e), active forms of Caspase3 and Caspase8 (apoptosis markers) (Supplementary Fig. S3f, g), and phosphorylation of MLKL, RIPK1, RIPK3 (necroptosis markers) (Supplementary Fig. S3h–j). Next, we examined PANoptosis in DOX-challenged heart tissues in mice. Consistent with the results in human heart tissues, DOX boosted PANoptosis in mouse hearts as evidenced by increased levels of AIM2, ZBP1, Pyrin (members of PANoptosome) (Fig. 5b–d), active forms of Caspase1 and GSDMD (pyroptosis markers) (Fig. 5e, f), active forms of Caspase3 and Caspase8 (apoptosis markers) (Fig. 5g, h), and phosphorylation of MLKL, RIPK1, RIPK3 (necroptosis markers) (Fig. 5i–k), the effects of which were aggravated by FUNDC1 ablation with little effect from FUNDC1 deficiency itself (Fig. 5b–k).

Co-immunoprecipitation (Co-IP) was performed to detect AIM2-PANoptosomes in AC-16 cardiomyocytes with AIM2 siRNA or scrambled siRNA transfection with or without DOX insult (Fig. 5l). ZBP1, AIM2, Pyrin, Caspase1, Caspase8, RIPK1, and RIPK3 were detected (without IgG identification) in RIPK3 immune complexes following DOX challenge in AC-16 but not control cardiomyocytes (Fig. 5l). AIM2 knockdown inhibited the formation of AIM2-PANoptosme, as evidenced by reduced ZBP1, AIM2, Pyrin, Caspase1, Caspase8, and RIPK1 pulled down by RIPK3 (Fig. 5l). Moreover, AIM2 knockdown using siRNA abolished elevated levels of ZBP1 and Pyrin following DOX challenge with or without FUNDC1 deficiency in AC-16 cardiomyocytes (Fig. 5m–o). These findings manifested that FUNDC1 deficiency promoted DOX-induced cardiomyocyte PANoptosis.

### FUNDC1 deficiency promoted DOX-induced cardiomyocyte PANoptosis in a mtDNA-dependent manner

**Fig. 6:**
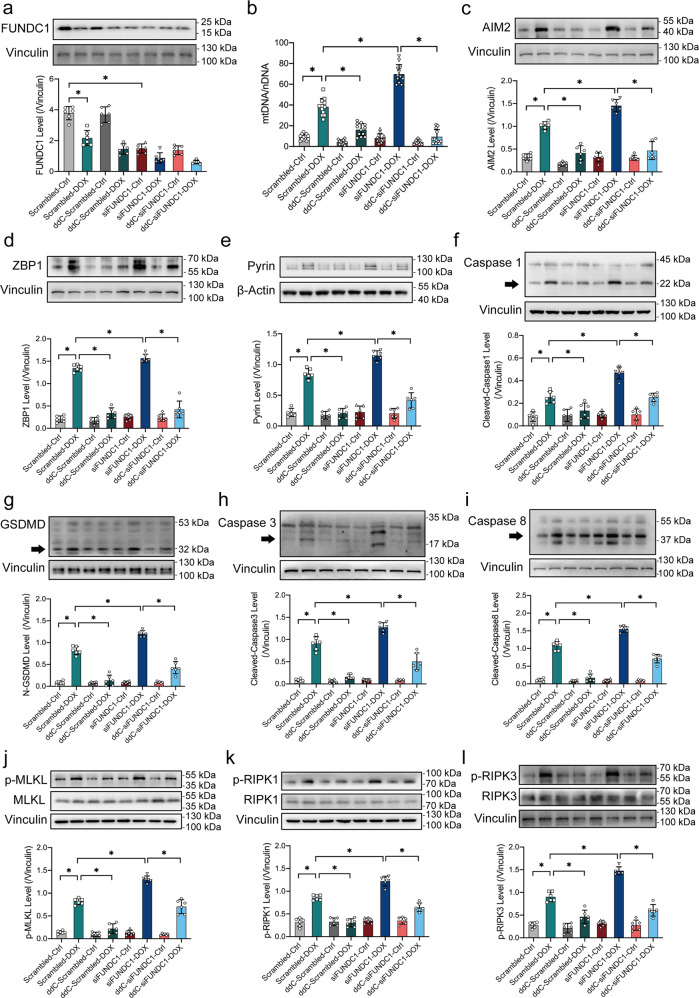
FUNDC1 deficiency promoted DOX-induced cardiomyocyte PANoptosis in a mtDNA-dependent manner. **a** Representative immunoblots and quantitative histograms of FUNDC1 (Vinculin as the loading control). **b** Ratio of mtDNA-to-nDNA in AC-16 cardiomyocytes exposed to DOX, under FUNDC1 deficiency with or without AIM2 ablation. **c**–**e** Representative immunoblots and quantitative histograms of AIM2, ZBP1, Pyrin (members of PANoptosome) (Vinculin or β-actin as the loading control). **f**, **g** Representative immunoblots and quantitative histograms of pyroptosis markers Caspase1 and GSDMD (Vinculin as the loading control). **h**, **i** Representative immunoblots and quantitative histograms of apoptosis markers Caspase3 and Caspase8 (Vinculin as the loading control). **j**–**l** Representative immunoblots and quantitative histograms of total and phosphorylation forms of MLKL, RIPK1, RIPK3 (necroptosis markers) (Vinculin as the loading control). Mean ± SEM, *n* = 12 (panel a) or 6 (rest of panels), *n* = 6 per group, *P < 0.05 between groups. One-way ANOVA followed by a Tukey’s test was used in (**a**–**l**).

To examine the role of mtDNA in the regulation of PANoptosis by FUNDC1 in DOX cardiotoxicity, nucleoside analog 2'3’-dideoxycytidine (ddC), a selective inhibitor of the mitochondrial DNA polymerase γ [36] was added to AC-16 cells that were transfected with scrambled or FUNDC1 siRNA transfection and treated with DOX challenge (Fig. 6a). As precited, ddC overtly decreased mtDNA level in cytoplasm regardless of the presence of DOX or not (Fig. 6b). Besides, DOX treatment provoked PANoptosis in AC-16 cardiomyocytes as evidenced by upregulated levels of AIM2, ZBP1, Pyrin (members of PANoptosome) (Fig. 6c–e), elevation in active forms of Caspase1 and GSDMD (pyroptosis markers) (Fig. 6f, g), upregulation in active forms of Caspase3 and Caspase8 (apoptosis markers) (Fig. 6h, i), and increased phosphorylation of MLKL, RIPK1, RIPK3 (necroptosis markers) (Fig. 6j–l), the effect of which was aggravated by FUNDC1 ablation. Interestingly, ddC treatment restored the FUNDC1 deficiency-elicited response to the level of scrambled-DOX treatment (Fig. 6c–l). These results suggested that FUNDC1 deficiency promoted DOX-induced cardiomyocyte PANoptosis in a mtDNA-dependent manner.

### FUNDC1 directly interacted with TUFM through its 96–133 amino acid fragment

**Fig. 7:**
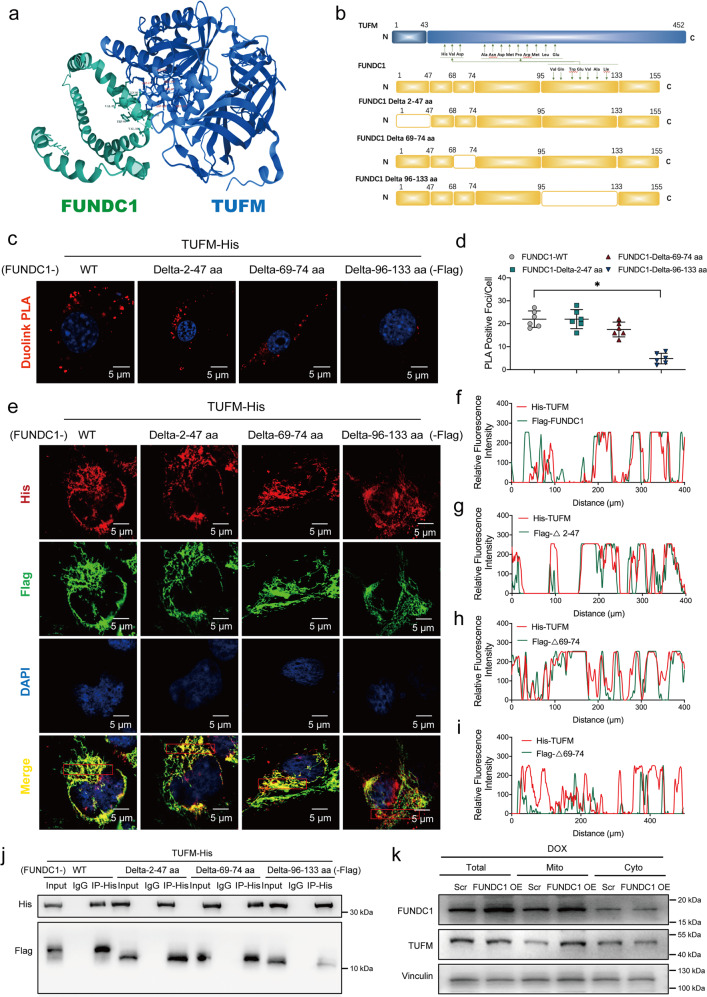
FUNDC1 directly interacted with TUFM. **a** Structure-based protein interaction interface analysis between FUNDC1 and TUFM. Cartoon represents predicted FUNDC1-TUFM complex structure, where interaction hotspot residues are labeled. **b** Schematic diagram of domain deletion of FUNDC1 for the following experiments. **c**, **d** Duolink PLA analysis of His-tagged TUFM and Flag-tagged FUNDC1 with or without different amino acid fragment deletion (FUNDC1 WT, FUNDC1 Delta-2–47 aa, FUNDC1 Delta-69–74 aa or FUNDC1 Delta-96–133 aa) in AC-16 cardiomyocytes (*n* = 6/group), scale bar = 5 μm. **e** Representative immunofluorescence staining images of co-localization of His-tagged TUFM (green) with Flag-tagged FUNDC1 (red) with or without different amino acid fragment deletion (FUNDC1 WT, FUNDC1 Delta-2–47 aa, FUNDC1 Delta-69–74 aa or FUNDC1 Delta-96–133 aa) in AC-16 cardiomyocytes, scale bar = 5 μm. **f**–**i** Fluorescence intensity curve of TUFM and FUNDC1. **j** Co-IP analysis of his-tagged TUFM and flag-tagged FUNDC1 with or without different amino acid fragment deletion (FUNDC1 WT, FUNDC1 Delta-2–47 aa, FUNDC1 Delta-69–74 aa or FUNDC1 Delta-96–133 aa) in AC-16 cardiomyocytes. **k** Representative immunoblots of FUNDC1 and TUFM in the mitochondrial or in the cytoplasm. RIPK3 (necroptosis markers) (Vinculin as the loading control). Mean ± SEM, *P < 0.05 between indicated groups, one-way ANOVA followed by a Tukey’s test was used in (**d**).

To discern possible interacting partners of FUNDC1, Mass spectrometry was performed following immunoprecipitation (IP-MS) in AC-16 cells transfected with FUNDC1 plasmids with Flag tag (FUNDC1-Flag) prior to immunoprecipitation with an anti-flag antibody. We identified that TUFM ranked atop among possible FUNDC1 binding proteins associated with mitochondria (Supplementary Table S2). PRISM tool analysis revealed that FUNDC1 and TUFM shared structural motif for direct binding. Prediction results from PRISM revealed that FUNDC1 and TUFM may form a protein interaction interface (Fig. 7a). To further explore specific interaction between FUNDC1 and TUFM, truncated mutants of FUNDC1 were constructed with deletion of 2–47 amino acid fragment (Delta-2–47 aa), 69–74 amino acid fragment (Delta-69–74 aa) or 96–133 amino acid fragment (Delta-96–133 aa) (Fig. 7b). WT and truncated variants of flag-tagged FUNDC1 plasmids were transfected with His-tagged TUFM plasmid in AC-16 cardiomyocytes. Duolink proximity ligation assay (PLA) detection assay revealed that WT and truncated variants of FUNDC1 interacted with TUFM with the exception of mutation of Delta-96–133 aa (Fig. 7c, d), suggesting an obligatory role for 96–133 aa domain of FUNDC1 in the direct interaction with TUFM. To explore the spatial relationship between FUNDC1 and TUFM, AC-16 cardiomyocytes were transfected with Flag-tagged WT or truncated variants of FUNDC1 together with His-tagged TUFM. In line with the Duolink PLA result, immunofluorescence staining demonstrated that WT and truncated variants of FUNDC1 were co-localized with TUFM with the exception of mutant Delta-96–133 aa (Fig. 7e–i). In addition, Co-IP assays also showed that WT and truncated variants of FUNDC1 interacted with TUFM, with the exception of mutant Delta-96–133 aa (Fig. 7j). These findings suggested that FUNDC1 directly interacted with TUFM through its 96–133 amino acid fragment.

Expression of TUFM was evaluated in heart tissues from WT and FUNDC1^−/−^ mice with or without DOX insult. Our data revealed that DOX downregulated the expression of TUFM, the effect of which was unaffected by FUNDC1 deficiency (Supplementary Fig. S4a). FUNDC1 may interact with TUFM in the cytoplasm or in mitochondria, thus impacting its transport and distribution. Next, to detect whether FUNDC1 affected the distribution of TUFM following DOX treatment, we examined levels of TUFM in mitochondria and cytosol in AC-16 cardiomyocytes, the result indicated that FUNDC1 overexpression overtly boosted mitochondrial TUFM content (Fig. 7k and Supplementary Fig. S4b). These data manifested that FUNDC1 recruited TUFM to promote its mitochondrial translocation.

### TUFM was responsible for FUNDC1-mediated mtDNA stabilization and inhibition of PANoptosis

**Fig. 8:**
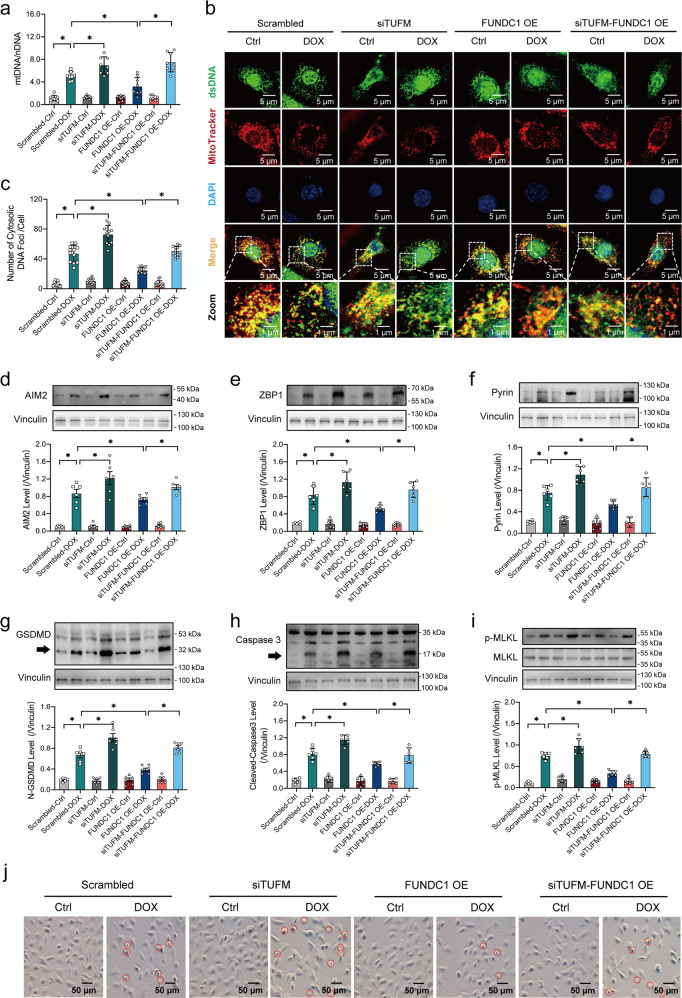
TUFM was responsible for FUNDC1-mediated mtDNA stabilization. **a** Ratio of mtDNA-to-nDNA in AC-16 cardiomyocytes exposed to DOX, under FUNDC1 overexpression with or without TUFM ablation. **b**, **c** Immunofluorescence staining images and quantitative analysis of mtDNA released to the cytoplasm (a portion of dsDNA not co-localized with MitoTracker or DAPI), scale bar = 5 μm, scale bar in zoom = 1 μm. **d**–**f** Representative immunoblots and quantitative histograms of AIM2, ZBP1, Pyrin (members of PANoptosome) (Vinculin as the loading control). **g** Representative immunoblot and quantitative histograms of GSDMD (Vinculin as the loading control). **h** Representative immunoblot and quantitative histogram of Caspase3 (Vinculin as the loading control). **i** Representative immunoblot and quantitative histogram of total and phosphorylated form of MLKL (Vinculin as the loading control). **j** Cell state observation (red circles denoting dead cells), scale bar = 50 μm. Mean ± SEM, *n* = 8 (panel **a**), 12 (panels **b**, **c**), and 6 (panels **d**–**i**), *P < 0.05 between indicated groups. One-way ANOVA followed by a Tukey’s test was used in (**a**, **c**, **d**–**i**).

To examine the role of TUFM in FUNDC1-elicited regulation of mtDNA and PANoptosis in DOX-induced cardiotoxicity, AC-16 cardiomyocytes were transfected with FUNDC1 plasmid, either alone or with TUFM siRNA, prior to DOX insult (Supplementary Fig. S5a, b). FUNDC1 overexpression was found to protect against DOX-induced mtDNA release, the effect of which was canceled off by TUFM deletion (Fig. 8a–c). Besides, FUNDC1 overexpression defended against DOX-provoked PANoptosis in AC-16 cardiomyocytes as evidenced by reversed levels of AIM2, ZBP1, Pyrin (members of PANoptosome) (Fig. 8d–f), active forms of Caspase1 and GSDMD (pyroptosis markers) (Fig. 8g and Supplementary Fig. S5c), and active forms of Caspase3 and Caspase8 (apoptosis markers) (Fig. 8h and Supplementary Fig. S5d), and phosphorylation of MLKL, RIPK1, RIPK3 (necroptosis markers) (Fig. 8i and Supplementary Fig. S5e, f). Intriguingly, FUNDC1-induced beneficial effects were also abolished by TUFM ablation with little effect from TUFM deficiency itself (Fig. 8d–i and Supplementary Fig. S5a–f). In addition, we examined AC-16 cells transfected with FUNDC1 plasmid, either alone or with TUFM siRNA, prior to DOX insult. We found that FUNDC1 overexpression protected against cell death (shrunken and brightened cells or the PI-positive cells), the effect of which was again annihilated by TUFM ablation (Fig. 8j and Supplementary Fig. S5g, h). Besides, TUFM knockdown exerted reminiscent responses in mtDNA release (Supplementary Fig. S6a) and upregulation of PANoptosis-related proteins in a manner reminiscent of DOX challenge (Supplementary Fig. S6b–i). These results suggested that TUFM was likely to be responsible for FUNDC1-mediated mtDNA stabilization and inhibition of PANoptosis in cardiomyocytes underneath DOX challenge.

## Discussion

Salient findings from our present study revealed that levels of FUNDC1 were significantly downregulated in heart tissues of patients with dilated cardiomyopathy (DCM), in heart tissues of mice with DOX insult, as well as in DOX-treated AC-16 cardiomyocytes. In DOX-induced murine model of cardiac injury, a downregulated expression of FUNDC1 was observed in heart tissues at 1, 2, 3, and 4-week following completion of DOX insult, with the most pronounced response at 1 week. In line with this observation, echocardiographic analysis showed that DOX challenge impaired fractional shortening of left ventricle at 1, 2, 3, and 4-week following completion of DOX challenge, with the most pronounced responsiveness at 1 week, suggesting a connection of downregulation of FUNDC1 with cardiac dysfunction in DOX challenge. We demonstrated that FUNDC1 deficiency decreased mouse survival (especially promoting initiation of death), as well as aggravated myocardial, cardiomyocyte contractile dysfunction, and cardiomyocyte mitochondrial injury following DOX challenge. Using qPCR and immunofluorescence staining techniques, FUNDC1 was found to fend off DOX-induced cardiomyocyte mtDNA release into cytoplasm, which then turn on dsDNA senser AIM2 to form AIM2-PANoptososme and boosted PANoptosis in cardiomyocytes. Further mechanistic study revealed that FUNDC1 directly interacted with TUFM through its 96–133 amino acid fragment as evidenced by Co-IP, Duolink PLA and immunofluorescence co-localization assays. Moreover, our data suggested that TUFM was likely responsible for FUNDC1-mediated mtDNA stabilization and PANoptosis inhibition in cardiomyocytes. These results collectively uncovered a protective role of FUNDC1 in DOX cardiotoxicity through inhibition of mtDNA release and PANoptosis in cardiomyocytes.

PANoptosis, the newly proposed programmed cell death pattern, is believed to provoke severe damage to cells, tissues, and organs [37, 38]. Nonetheless, it remains elusive with regards to the role of PANoptosis in cardiovascular diseases in particular DOX cardiotoxicity. Here, through the detection of PANoptosis-related proteins and the formation of PANoptosomes in heart tissues of patients with DCM and DOX-challenged mice, DOX was found to evoke PANoptosis in cardiomyocytes, which shed some lights towards a novel mechanism of DOX cardiotoxicity. Although studies have revealed that DOX challenge can lead to pyroptosis [39], apoptosis [40, 41], and necroptosis [42] in cardiomyocytes, although little is known with regards to the crosstalk and coordination among these three cell death patterns. It has been conceptualized that a flexible skeleton is formed in PANoptosome during the process of PANoptosis, with core components of the three cell death patterns being recruited to provoke pyroptosis, apoptosis and necroptosis concurrently to jointly initiate PANoptosis [27, 28]. In addition, previous studies were mainly focused on the resistant role of PANoptosis in immune response to virus infection [43]. Findings from our study indicate that mtDNA release may serve as another trigger of AIM2-PANoptosome and PANoptosis in pathological conditions, thus providing more insights into the activation of PANoptosis.

In this study, TUFM was identified as an interacted partner of FUNDC1. TUFM is a nuclear gene-encoded mitochondrial protein widely expressed in various tissues including the heart [44]. TUFM is essential for mitochondrial respiratory function in addition to its role as a key factor for translation of mitochondrial genes in the governance of amino acid elongation of mtDNA-encoded peptides [34]. Besides, TUFM also plays a cardinal role in the nucleotide excision repair of mtDNA [35]. Moreover, as a mitochondrion-cytosol dual-localized protein, a majority of TUFM is transported to mitochondria following the completion of synthesis [45]. Thus, it is possible that FUNDC1 can interact with TUFM in the cytoplasm to promote its transportation into mitochondria. On the other side of the coin, FUNDC1 may also recruit TUFM in mitochondria to pin it within the cytoplasm, creating a dynamic two-way trafficking modality. Interestingly, our data suggested that FUNDC1 did not bind TUFM through its inner-mitochondrial domain, but rather through its 96–133 amino acid fragment, an outer-mitochondrial domain of FUNDC1. These findings would collectively favor the notion that FUNDC1 recruits TUFM to promote its mitochondrial translocation as the mainstream direction of TUFM transport, which is supported by the increased mitochondrial content of TUFM.

FUNDC1 is a mitophagy receptor regulating chronic pathological progresses including heart diseases [21, 23, 46–48]. Our results also suggested the role of FUNDC1 as a protective regulatory factor in DOX-induced heart injury through fending off mtDNA damage and release. A protective role of FUNDC1 for mtDNA has been proposed, mainly through its function of mediating mitophagy and mitochondrial dynamics [49–51]. In our hands, we unveiled that FUNDC1 helped to defend against mtDNA damage by recruiting a mtDNA-repair protein TUFM and promoting its mitochondrial translocation. Besides, another possible explanation for FUNDC1-ameliorated mtDNA damage and cytoplasmic release is through regulation of mitochondrial damage including mitochondrial membrane potential collapse and ROS production. These results have provided some new insights for the modality of FUNDC1 in mitochondrial quality control beyond mitophagy.

Potential novel strategies were reported recently to fend off DOX-induced cardiotoxicity. Resveratrol was deemed to display cardioprotective and anticancer effects, through regulating the p62-NRF2 axis to inhibit ferroptosis in DOX-treated cardiomyocytes [52]. A miR-4732-3p mimic was also proved to be cardioprotective in cardiac and fibroblast cultures following DOX challenge, via regulating genes of TGFβ and Hippo signaling pathways [53]. Moreover, vinpocetine, a phosphodiesterase inhibitor, was found to guard against DOX-induced cardiotoxicity as manifested by a significant decrease of cardiac enzymes, HIF-1α and TNF-α, via modulation of HIF/VEGF and cGMP/cAMP/SIRT signaling pathways [54]. Nicotinamide riboside administration was shown to exert a cardioprotective property against chronic DOX-induced cardiomyopathy [55]. These findings provide new ways of DOX-induced cardiomyopathy prevention and treatment approaches. Findings from our study provided compelling evidence that FUNDC1 protects against DOX cardiotoxicity and mitochondrial injury via regulation of cardiomyocyte PANoptosis. Mechanically, FUNDC1 restrains mtDNA release to the cytoplasm by direct binding and recruiting TUFM to mitochondria to fend off mtDNA damage and mtDNA cytosolic release, thus preventing formation of PANoptosome and PANoptosis during the DOX challenge. Nonetheless, future study is needed to examine such PANoptosis mechanism in the realm of cancer chemotherapy in various tumors, to offer guidance with regards to the impact of FUNDC1 on cancer therapy. Considering the limited strategies to combat cardiotoxicity of DOX, induction and boosting of FUNDC1 (maybe through gene intervention including AAV administration, FUNDC1 activator such as empagliflozin [56], or other specific activators of FUNDC1) may be a promising new strategy in the therapeutics of DOX cardiotoxicity.

## Materials and methods

### Human samples

Human heart samples were obtained from patients who have undergone heart transplant surgery due to DCM or the doner heart failed with transplantation due to non-cardiac reasons (*n* = 6/group, informed consent was obtained from all subjects), from the Sun Yat-Sen Memorial Hospital, Sun Yat-Sen University (Guangzhou, China). The study was approved by the institutional ethics committee of Sun Yat-Sen Memorial Hospital.

### Experimental animals and DOX challenge

All animal procedures were approved by the Institutional Animal Care and Use Committee at the Zhongshan Hospital Fudan University (Shanghai, China). Global FUNDC1 knockout (FUNDC1^−/−^) mice were generated as described in our earlier report [21]. Male WT and FUNDC1^−/−^ mice (6 to 8-week-old) were randomly subjected to DOX challenge (5 mg/kg, i.p., four doses, once per week).

### Echocardiographic assessment

Following DOX challenge (for 4 weeks), mice were anesthetized with isoflurane (1–2%) prior to the M-mode echocardiographic assessment. A two-dimensional (2D) guided M-mode echocardiography equipped with a 22–55 MHz transducer (MS550D, FUJIFILM VisualSonics, Toronto, ON, Canada) was applied to measure cardiac geometry and function (Vevo 2100, FUJIFILM Visualsonics) according to published procedures [57, 58].

### Histological examination

Following anesthesia, mouse hearts were arrested in diastole in 10% KCl solution, and were exercised and maintained in 10% neutral-buffered formalin for 24 h at room temperature. Specimens were embedded in paraffin, cut into 5-μm sections and stained with Masson trichrome. The percentage of fibrosis was calculated using a digital microscope (×400) and the Image J (version 1.34S) software.

### Transmission electron microscopy (TEM)

Cubic heart pieces were dissected and immersed with 2.5% glutaraldehyde in 0.1 M sodium phosphate (pH 7.4) for at least 24 h at 4 °C. Tissues were dehydrated through graded alcohols and were embedded in Epon Araldite prior to fixation in 1% OsO_4_ for 1 h. Ultrathin sections (75–80 nm) were produced using an ultramicrotome (Leica, Wetzlar, Germany) equipped with a Diatome diamond knife, and were stained with uranyl acetate for 10 min and lead citrate for another 5 min. Specimens were visualized under an 40–120 kV transmission electron microscope (Hitachi H600 Electron Microscope, Hitachi, Japan. Images were captured using the Digital Micrograph software.

### Cell culture and treatment

Adult mouse cardiomyocytes (AMCMs) were isolated as described [57, 59, 60]. In brief, adult male C57/BL6J mice (8 to 10-week-old) were anesthetized, prior to the opening of the chest cavity to fully expose the heart. The inferior vena cava and descending aorta were removed before injection of an EDTA buffer into the right ventricle. Hearts were removed and perfused with a collagenase buffer (type II and IV collagenase). When tissues turned slightly pale and flaccid, left ventricles were minced into 1-mm^3^ pieces in a stop buffer. Extracellular Ca^2+^ levels were slowly restored to 1.2 mM. A yield of at least 80% rod-shaped CMs was deemed successful [59, 60].

Human AC-16 cardiomyocytes recently authenticated were cultured in a Dulbecco’s Modified Eagle Medium (DMEM, Gibco, Waltham, ME, USA), containing 10% fetal bovine serum albumin (FBS, Gibco) at 37 °C, 5% CO_2_. AC-16 cardiomyocytes were treated with DOX (1 μM, 24 h, Sigma Aldrich, MO, USA). To determine the involvement of mtDNA, a nucleoside analog 2'3’-dideoxycytidine (ddC, Selleck Chemicals, Houston, TX, USA, 40 μg/mL for 5 days) was added to AC-16 cells when cells grow to a proper density for further experimentation.

### Shortening/re-lengthening and intracellular Ca^2+^ recording of AMCMs

Mechanical properties of cardiomyocytes were assessed using a Softedge MyoCam system (IonOptix Corporation, Milton, MA, USA) equipped with an IX-70 Olympus inverted microscope. Cardiomyocytes were electrically stimulated at 0.5 Hz in a contractile buffer containing NaCl 135 mM, KCl 4.0 mM, CaCl2 1.0 mM, MgCl 1.0 mM, glucose 10 mM and HEPES 10 mM. Cell shortening was assessed including peak shortening (PS), maximal velocity of shortening (+dL/dt), maximal velocity of re-lengthening (−dL/dt), time-to-PS (TPS), and time-to-90% re-lengthening (TR_90_). For intracellular Ca^2+^ recording, cardiomyocytes were loaded with Fura-2/AM (0.5 μM) for 10 min, and fluorescence measurements were recorded with a dual-excitation fluorescence photomultiplier system (IonOptix). To assess intracellular Ca^2+^ signaling, cells were exposed to light emitted by a 75-W lamp and passed through 360 nm or a 380 nm filter, while being stimulated to contract at 0.5 Hz. Fluorescence emissions were detected between 480 and 520 nm and the alterations in fura-2 fluorescence intensity (FFI) were quantitated from the FFI ratio at 360 nm to 380 nm. Fluorescence decay time was assessed as an indicator of intracellular Ca^2+^ clearing [57, 61].

### Plasmid construction and transfection

Full-length and domain truncated FUNDC1 plasmids (FUNDC1 Delta-2-47aa, FUNDC1 Delta-69-74aa, and FUNDC1 Delta-96–133) tagged with Flag were cloned in pcDNA3.1 + (Dongxuan Genes, Kunshan, China). TUFM tagged with His and negative controls were cloned in pcDNA3.1 + (Dongxuan Genes, Kunshan, China). Plasmids were transfected into AC-16 cardiomyocytes following incubation for 48 h.

### Mitochondrial respiration measurement

Mitochondrial respiration was measured by analyzing mitochondrial oxygen consumption rate (OCR) using a Seahorse XFe96 analyzer (Seahorse Biosciences, North Billerica, MA, USA) [62]. In brief, cells were seeded at 40,000 cells/well on 96-well XFe96 cell culture microplates and then treated as designed. For respiration assays, cells were exposed to DOX for 24 h, and OCR was measured every 3 min over 90 min. First, OCR was quantified in basal condition (20 mmol/L glucose), 1 μM oligomycin (ATP synthase inhibitor), then 0.125 μM FCCP (mitochondrial respiration uncoupler), and finally with 1 μM rotenone/antimycin A (complex I and III inhibitors, respectively). Seahorse XF-24 software was used to calculate the OCR automatically.

### Cytosolic mtDNA isolation

Following lysis, AC-16 cells were centrifuged (700 × g, for 10 min, at 4 °C) to remove the nuclei. Then the supernatant volume was normalized according to protein concentration. Next, cell lysate was centrifuged (10,000×*g*, for 30 min, at 4 °C) again to isolate the cytosolic fraction (including mtDNA and nDNA). mtDNA was detected using quantitative PCR with the gene sequences coding for mitochondrial cytochrome c oxidase 1 (mtCOI) as primers. Nuclear DNA was measured using sequences coding 18 S ribosomal RNA as primers [63]. Finally, the ratio of mtDNA copy number to nDNA copy number was calculated to assess the release of mtDNA into the cytoplasm. The primers for human mtCOI and 18 S rDNA are listed in Supplementary Table S1 (Dongxuan Genes, Kunshan, China).

### Real-time quantitative PCR

Trizol Reagent (Invitrogen, NY, Empire State, USA) was used to extract total RNA. Then purity and concentration of RNA were determined using a NanoDrop 2000 spectrophotometer (Thermo Fisher Scientific, Waltham, Maine state, USA). PrimeScriptTM RT Master Mix (Takara, Shiga, Japan) was used to conduct reverse transcription for the synthesis of cDNA. Real-time quantitative PCR was performed using FastStart Essential DNA Green Master (Roche, Shanghai, China). GAPDH was employed as the reference gene. Relative gene expression was calculated using 2-ddCt method. The primers are listed in Supplementary Table S1 (Dongxuan Genes, Kunshan, China).

### Isolation of mitochondria

Preparation of mitochondria was conducted using the mitochondria/cytosol fractionation kit (Abcam, #ab65320). Isolation of mitochondria was carried out on ice to prepare the mitochondrial and cytosolic fractions for western blot analysis.

### Western blot analysis

Heart tissues and cells were homogenized and sonicated in a RIPA lysis buffer (Beyotime, Shanghai, China), including a protease inhibitor cocktail. Target proteins were separated by SDS/PAGE gels, before being transferred onto 0.22-μm PVDF membranes. After blocking with 5% bovine serum albumin (BSA), PVDF membranes were incubated with primary antibodies, including anti-Vinculin (Abcam, #ab219649), anti-FUNDC1(Abcam, #ab224722), anti-AIM2 (Abcam, #ab204995), anti-ZBP1 (Abcam, #ab290736), anti-Pyrin (Abcam, #ab195975), anti-Caspase1 (Abcam, #ab207802), anti-Caspase3 (Abcam, #ab32351), anti-Caspase8 (Proteintech, 13423-1-AP), anti-GSDMD (Abcam, #ab209845), anti-MLKL (Abcam, #ab184718), anti-p-MLKL (Abcam, #ab196436) anti-RIPK1 (Cell Signaling Technology, #73271), anti-p-RIPK1 (Cell Signaling Technology, #44590), anti-RIPK3 (Cell Signaling Technology, #10188), anti-p-RIPK3 (Cell Signaling Technology, #93654), anti-p-TUFM antibodies (Abcam, #ab173300) and anti-Flag antibodies (Cell Signaling Technology, #14793) overnight at 4 °C. Secondary antibodies were employed for membrane incubation. Films were scanned and detected with a Bio-Rad calibrated densitometer.

### Structure-based protein interaction interface analysis between FUNDC1 and TUFM

The protein structure of FUNDC1 was predicted by template-based homology structure modeling tool SWISS-MODEL (https://www.swissmodel.expasy.org), using PDB structure 3BK6, chain A (covering residues 74-256, sequence identity = 21.64%), and 2IP6, chain A (covering residues 82–131, sequence identity = 10.00%), as the template, respectively. The structure of TUFM was downloaded from the PDB database (PDB ID:7A5G). Prediction of the potential interaction interface between TUFM and FUNDC1 was obtained from PRISM tool (http://cosbi.ku.edu.tr/prism). Prediction results were visualized using the PyMol tool (http://pymol.org).

### Co-immunoprecipitation (Co-IP)

Total protein was extracted from AC-16 cells transfected with plasmids as designed using NP-40 lysis buffer. 50 μL total protein was extracted as input. Then, protein samples were exposed to the primary antibody of His (Abcam, #ab18184) or isotype control immunoglobulin G (Cell Signaling Technology, USA, #3900 S) at 4 °C overnight. 20 mL protein A/G agarose beads (Sea Biotech, China, #P001-2) were added into the mixture, and incubated for 3 h at 4 °C with rotation. Centrifuged the cell lysate (10,000×*g* for 30 min at 4 °C) to collect protein A/G agarose beads, and washed the beads 3–5 times with NP-40 lysis buffer. Finally, proteins were analyzed by western blotting.

### Immunoprecipitation assay and mass spectrometry

AC-16 cells were transfected with FUNDC1-Flag plasmids following incubation for 48 h. Cell lysates were immunoprecipitated with the anti-Flag magnetic beads (Cell Signaling Technology, #82103). Precipitates were separated by SDS-PAGE, and were then stained with Coomassie blue. Subsequently, protein bands were cut into small pieces and were subjected in-gel digestion. Finally, the extracted peptides from the gel pieces were prepared for proteomic data analysis using liquid chromatography-tandem mass spectrometry (LC-MS/MS).

### Immunofluorescence staining

Cells were fixed by 4% paraformaldehyde for 10 min, were then permeabilized and blocked for 1 h prior to incubation with specified antibodies (dsDNA, Abcam, #ab27156, His, Abcam, #ab18184, and Flag, Abcam, #ab205606) overnight at 4 C. Cells were incubated with the corresponding secondary antibodies conjugated with Alexa Fluor (Cell Signaling Technology, Boston, USA) for 2 h in the dark. After staining, immunofluorescence was examined using a laser confocal microscope with a ×630 oil immersion objective with 488 and 561 nm laser excitation (Leica, Wetzlar, Germany). Results were analyzed using a coloc2 plug-in (Fiji, version 2.0, Rawak Software Inc., Stuttgart, Germany) of Image J software.

### Propidium iodide (PI)/calcein staining

Cells were incubated with the Calcein AM/PI fluorescent dye (Beyotime, Shanghai, China) at 37 °C for 30 min. Then, cells were washed with a warm phosphate-buffered saline (PBS) buffer three times prior to visualization under a fluorescence microscope.

### Measurement of mitochondrial ROS (mtROS)

Cells were incubated with the MitoSox Red fluorescent dye (5 μM, Thermo Fisher, Waltham, Maine state, USA) at 37 °C for 30 min. Next, cells were washed with warm PBS buffer for three times. Finally, cells were observed under a Leica confocal microscopy (Wetzlar, Germany).

### Measurement of mitochondrial membrane potential (MMP)

Mitochondrial membrane potential (MMP) was evaluated using tetramethylrhodamine methyl Ester (TMRM) staining, and mitochondrial morphology was assessed using MitoTracker staining. Cells were stained with TMRM (20 nM, Molecular Probes, Invitrogen, Invitrogen, Carlsbad, California, USA) or MitoTracker Red solution (20 nM, Molecular Probes, Invitrogen, Carlsbad, California, USA) for 30 min at 37 °C. Cells were then washed with warm PBS buffer for three times. Cells were observed using a fluorescence microscope. Image J was used to evaluate red fluorescence intensity.

### Statistical analysis

All quantitative data were presented as mean ± standard error of the mean (SEM). Results were analyzed using a Prism 8.0 software (GraphPad, San Diego, CA). Comparison between two groups were conducted using the Student’s *t* test (two-tailed). Comparison among multiple groups were conducted using one-way ANOVA followed by Tukey’s test for post hoc analysis. The survival rate among multiple groups was conducted using the log-rank test. Statistical significance was set at *P* < 0.05.

## Supplementary information

supplemental materials

Figure s1

Figure s2

Figure s3

Figure s4

Figure s5

Figure s6

Figure s7

western blots

aj-checklist

## Data Availability

The authors confirm that the data supporting the findings of this study are available within the article or its supplementary materials.
