# Supplementary material for: FUNDC1 protects against doxorubicin-induced cardiomyocyte PANoptosis through stabilizing mtDNA via interaction with TUFM
Source: Cell Death Dis. 2022 Dec 5;13(12):1020. doi: 10.1038/s41419-022-05460-x (PMC9723119; doi:10.1038/s41419-022-05460-x)
Supplement: Supplementary file 1 — supplemental materials [file 41419_2022_5460_MOESM1_ESM.docx]

**SUPPLEMENTAL MATERIALS**

**FUNDC1 Protect against Doxorubicin induced Cardiomyocyte PANoptosis through Stabilizing mtDNA via Interaction with TUMF**

Yaguang Bi^1,2#^, Haixia Xu^1,3#^, Xiang Wang^1,2#^, Hong Zhu^4^, Junbo Ge^1,2^*, Jun Ren^1,2,5^* and Yingmei Zhang^1,2^*

**^1^****Department of Cardiology, Zhongshan Hospital, Fudan University, Shanghai Institute of Cardiovascular Diseases, Shanghai, China; ^2^National Clinical Research Center for Interventional Medicine, Shanghai 200032, China; ^3^Department of Cardiology, Affiliated Hospital of Nantong University, Nantong 226001, China; ^4^Laboratory of Oral Microbiota and Systemic Diseases, Shanghai Ninth People's Hospital, College of Stomatology, Shanghai Jiao Tong University School of Medicine, Shanghai 200125, China; ^5^Department of Laboratory Medicine and Pathology, University of Washington, Seattle, WA 98195 USA**

**^#^Equal contribution**

**Running title:** FUNDC1 and PANoptosis in doxorubicin cardiomyopathy

**Data availability statement**: The authors confirm that the data supporting the findings of this study are available within the article or its supplementary materials.

***Correspondence to:** Prof. Yingmei Zhang (zhang.yingmei@zs-hospital.sh.cn) or Prof. Jun Ren ([jren@uw.edu](mailto:jren@uw.edu)) or Prof. Junbo Ge (jbge@zs-hospital.sh.cn)

**SUPPLEMENTAL TABLE**

**Table S1.** **Primer sequence of 18S rDNA, mtCOI and GAPDH used in our experiments**

| Gene name | Forward primer，5’-3’ | Reverse primer，3’-5’ |
| --- | --- | --- |
| 18S rDNA (nDNA) | TAGAGGGACAAGTGGCGTTC | CGCTGAGCCAGTCAG TGT |
| mtCOI (mtDNA) | GCCCCCGATATGGCGTTT | GTTCAACCTGTTCCTGCTCC |
| GAPDH (Mouse) | ACCACAGTCCATGCCATCAC | TCCACCACCCTGTTGCTGTA |

**Table S2.** **FUNDC1-interacting proteins identified by IP-MS analysis (top ten mitochondrial proteins).**

| **Protein** | **Score** | **Coverage** | **Peptides** |
| --- | --- | --- | --- |
| **TUFM** | 1573.06 | 45.35 | 17 |
| **HSPD1** | 1464.55 | 48.87 | 22 |
| **SLC25A3** | 1395.41 | 32.49 | 14 |
| **DLST** | 1343.01 | 27.75 | 10 |
| **DLAT** | 1284.59 | 27.41 | 14 |
| **ATP5F1** | 1185.81 | 50.39 | 20 |
| **SUCLG1** | 1181.8 | 30.64 | 9 |
| **SLC25A12** | 1177.27 | 33.23 | 17 |

**SUPPLEMENTAL FIGURES**

**
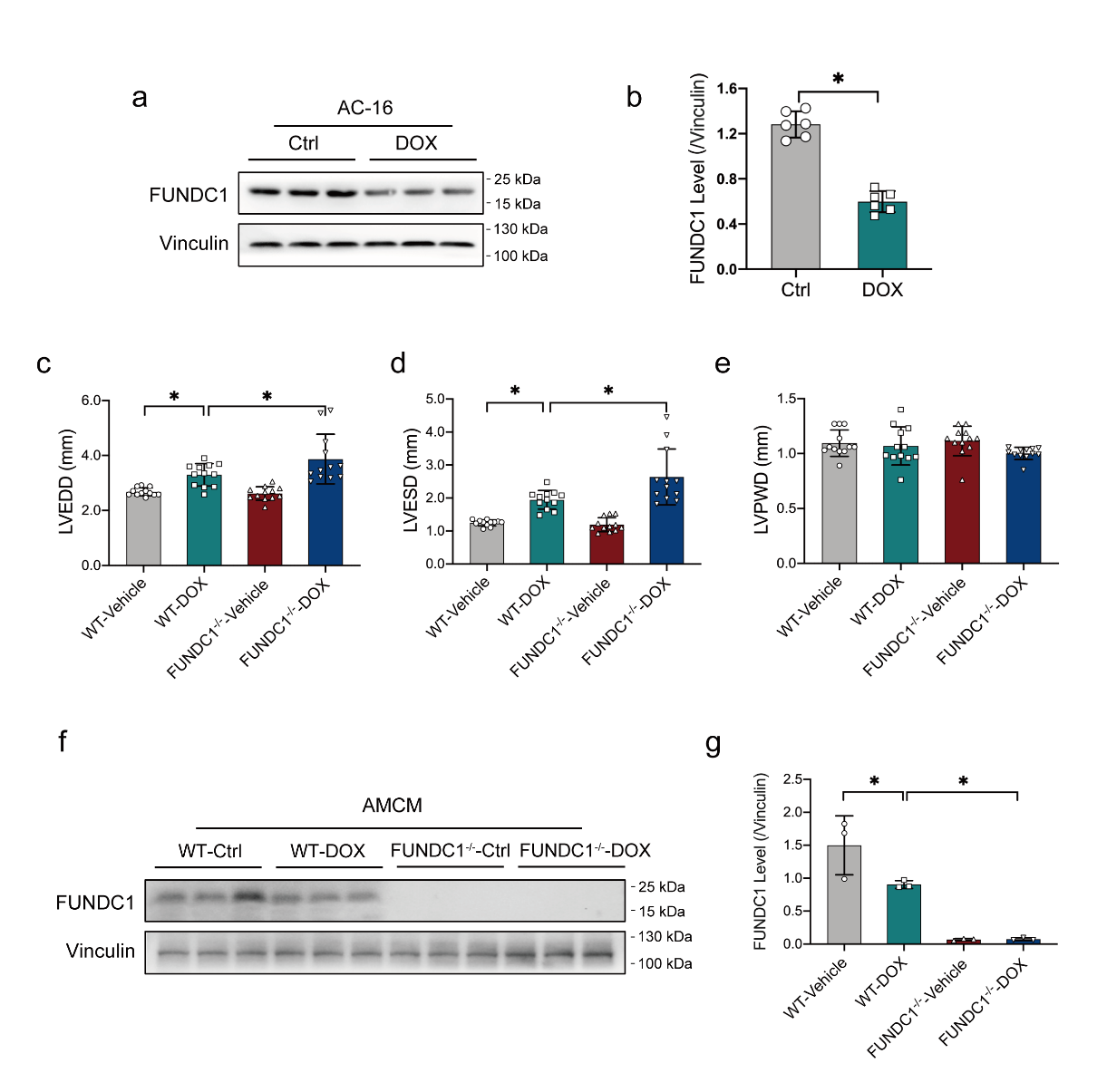
****Supplemental Fig.S1** (a-b) Representative immunoblot and quantitative histogram of FUNDC1 in AC-16 cardiomyocytes with or without DOX treatment (Vinculin as the loading control) (n = 6/group); (c-e) Echocardiographic analysis of left ventricular end-diastolic diameter (LVEDD), left ventricular end-systolic dimension (LVESD) and left ventricular posterior wall diameter in diastole (LVPWD) in heart tissues of mice at 1 W following Dox-modeling completion; (f-g) Representative immunoblot and quantitative histogram of FUNDC1 in AMCMs isolated from WT or FUNDC1^-/-^ mice with or without DOX challenge (Vinculin as the loading control) (n = 3/group). Mean ± SEM, **p* < 0.05，n = 6/group. student’s *t-*test was used in (b), one-way ANOVA followed Tukey's test was used in (c-e, g).

**
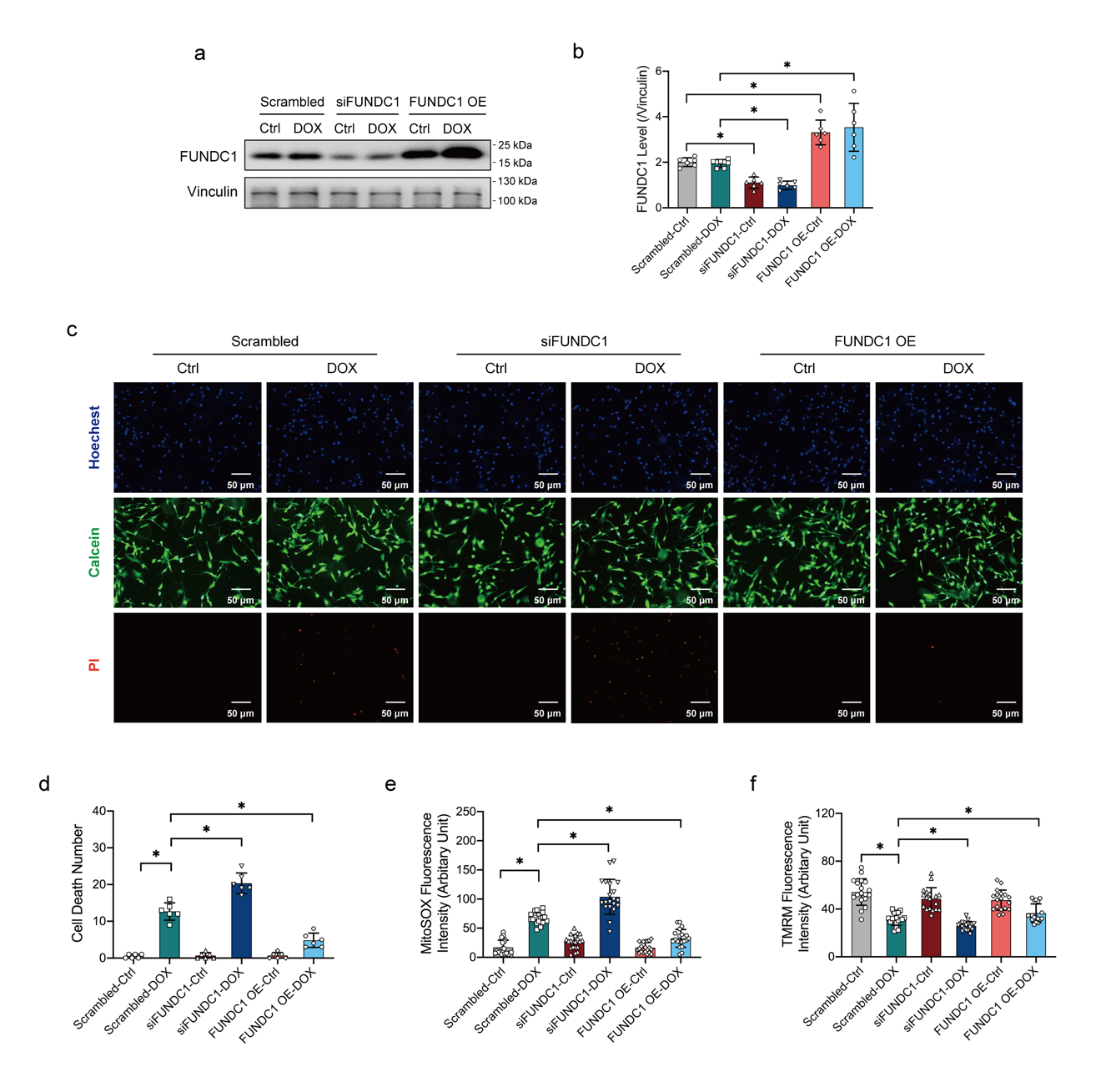
**

**Supplemental Fig.S2** (a-b) Representative immunoblot and quantitative histogram of FUNDC1 in AC-16 cardiomyocytes with FUNDC1 deletion or overexpression in the presence or absence of DOX challenge (Vinculin as the loading control) (n = 6/group); (c) PI/Calcein staining indicating cell death/live cell; (d) Quantitative analysis of cell death number (n = 6/group); (d) Quantitative analysis of MitoSOX staining, scale bar = 25 μm; (e) Quantitative analysis of TMRM staining, scale bar = 25 μm. Mean ± SEM, **p* < 0.05, n = 20/group. One-way ANOVA followed by a Tukey's test was used in (b-e).

**
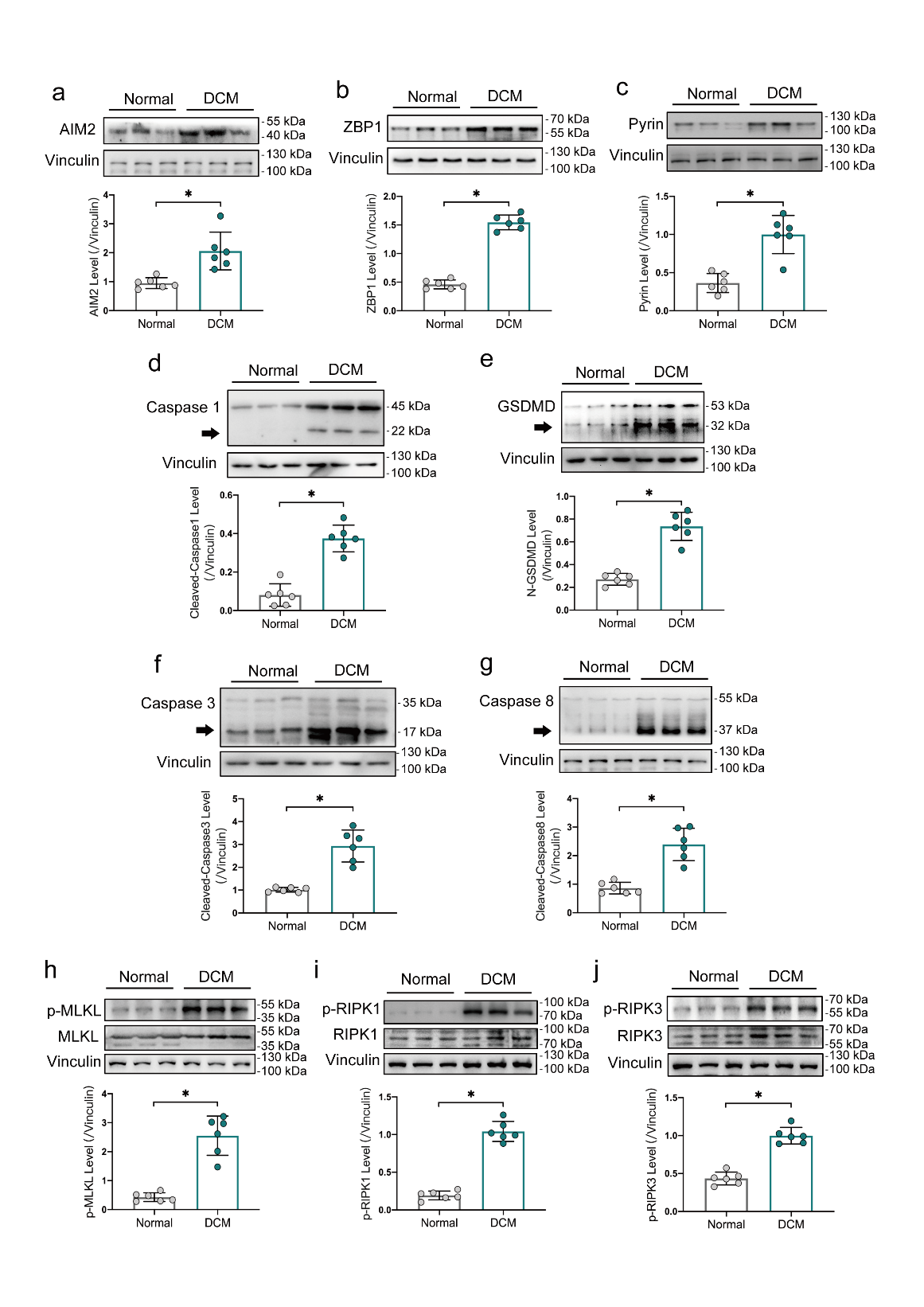
**

**Supplemental Fig.S3** (a-c) Representative immunoblots and quantitative histograms of AIM2, ZBP1, Pyrin (members of PANoptosome) in heart tissues of patients with DCM; (d-e) Representative immunoblots and quantitative histograms of Caspase1 and GSDMD (pyroptosis markers); (f-g) Representative immunoblots and quantitative histograms of Caspase3 and Caspase8 (apoptosis markers); (h-j) Representative immunoblots and quantitative histograms of total and phosphorylation forms of MLKL, RIPK1, RIPK3 (necroptosis markers). Vinculin as the loading control. Means ± SEM, **p* < 0.05, n = 6/group. Student’s *t-*test was used in (a-j).


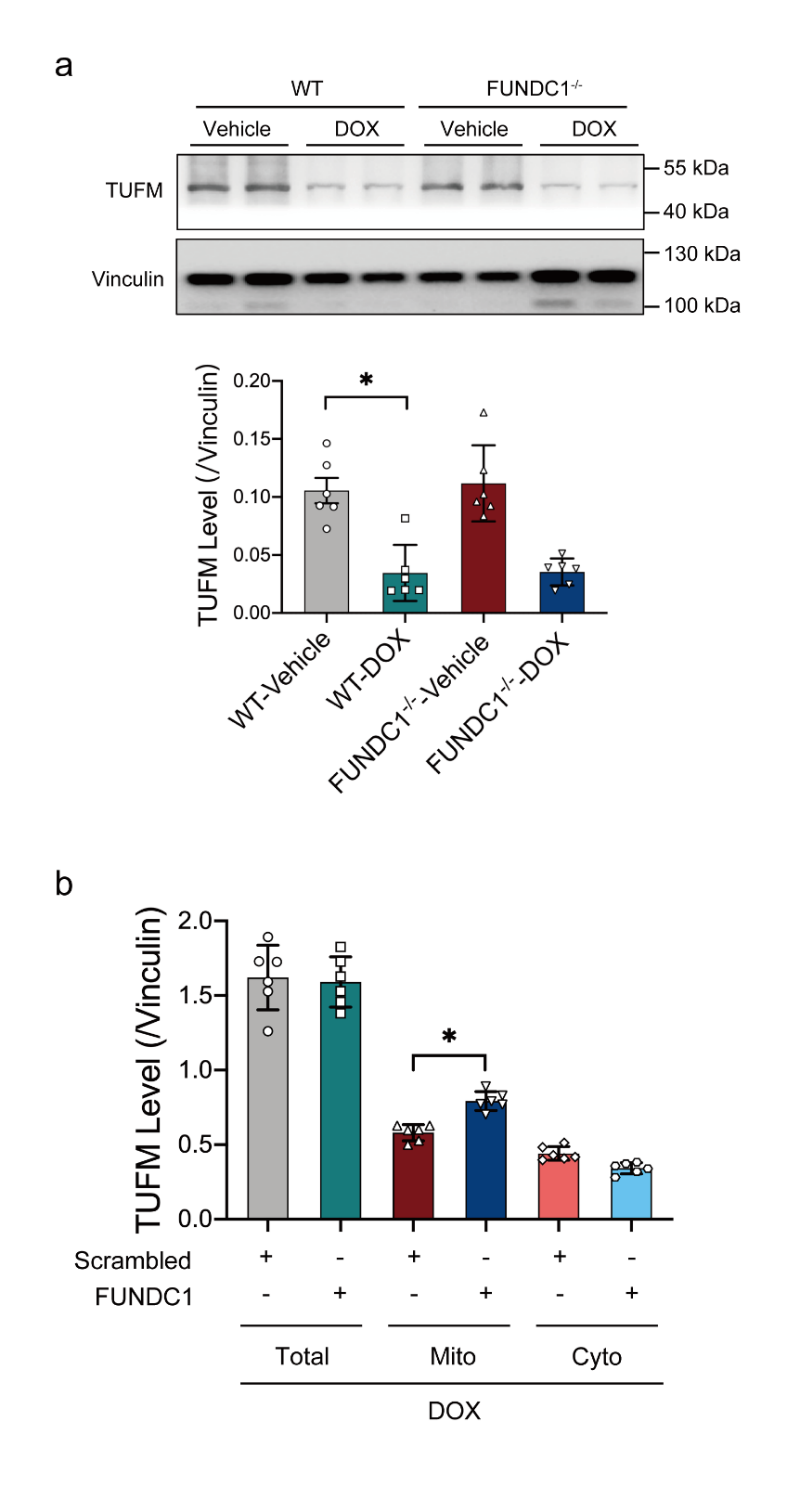


**Supplemental Fig.S4** (a) Representative immunoblot and quantitative histogram of TUFM in heart tissues of mice (Vinculin as the loading control); (b) Quantitative histogram of total, mitochondrial and cytosolic TUFM levels. Mean ± SEM, **p* < 0.05, n = 6/group. One-way ANOVA followed by a Tukey's test was used in (a-b).


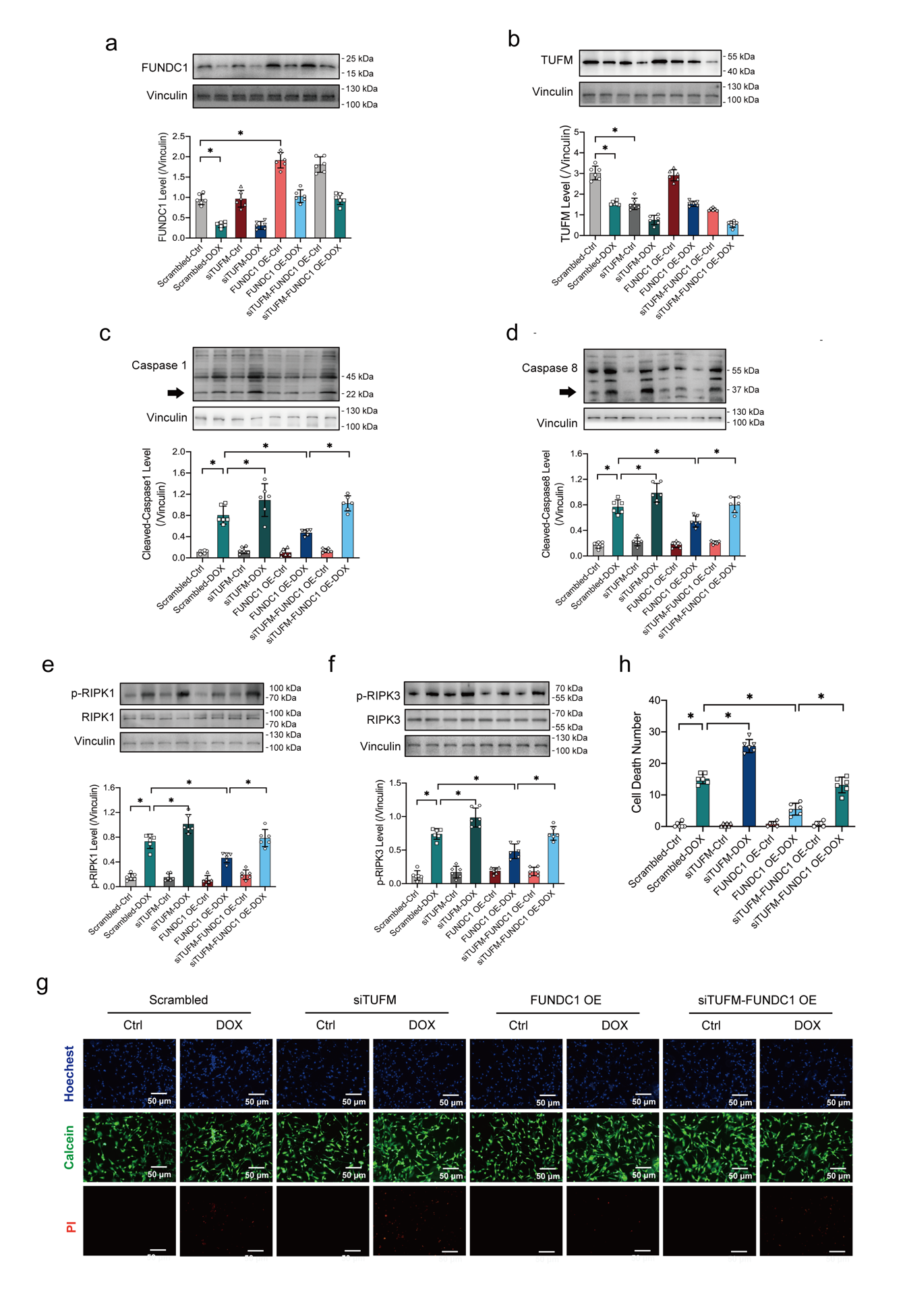


**Supplemental Fig.S5** (a) Representative immunoblots and quantitative histograms of FUNDC1 in AC-16 cardiomyocytes exposed to DOX, under FUNDC1 overexpression with or without TUFM ablation; (b) Representative immunoblots and quantitative histograms of TUFM; (c) Representative immunoblots and quantitative histograms of Caspase1; (d) Representative immunoblots and quantitative histograms of Caspase8; (e-f) Representative immunoblots and quantitative histograms of total and phosphorylation form of RIPK1 and RIPK3; (g) PI/Calcein staining indicating cell death/live cell; (h) Quantitative analysis of cell death number. Vinculin as the loading control in above panels. Mean ± SEM, **p* < 0.05, n = 6/group, one-way ANOVA followed by a Tukey's test was used in (a-g).


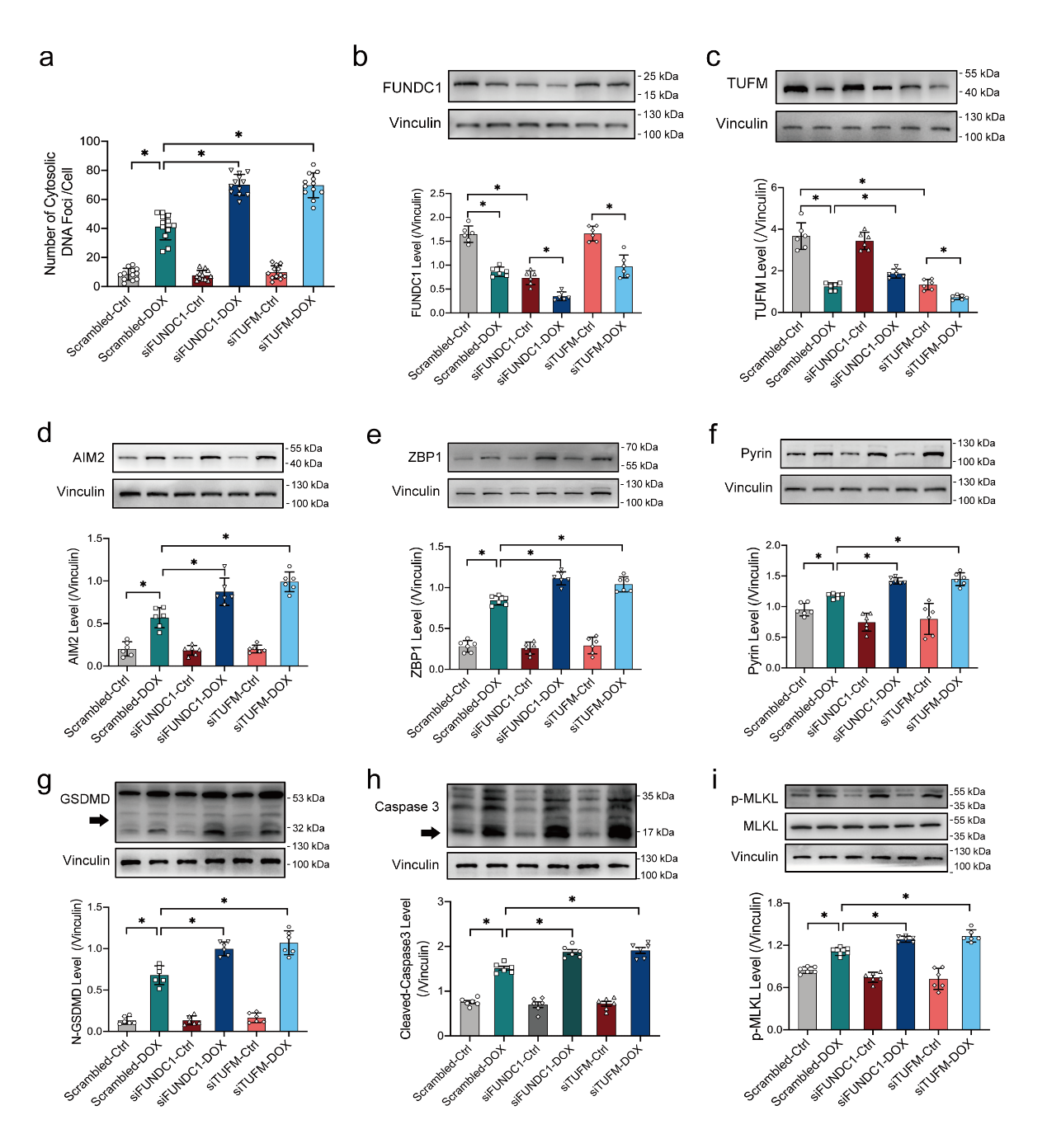


**Supplemental Fig.S6** (a) Ratio of mtDNA-to-nDNA in AC-16 cardiomyocytes exposed to DOX, with or without FUNDC1 or TUFM ablation; (b) Representative immunoblots and quantitative histograms of FUNDC1. (c) Representative immunoblots and quantitative histograms of TUFM. (d-f) Representative immunoblots and quantitative histograms of AIM2, ZBP1, Pyrin (members of PANoptosome); (g) Representative immunoblot and quantitative histograms of GSDMD; (h) Representative immunoblot and quantitative histogram of Caspase3; (i) Representative immunoblot and quantitative histogram of total and phosphorylated form of MLKL. Vinculin as the loading control. Mean ± SEM, **p* < 0.05, n = 6/group, one-way ANOVA followed by a Tukey's test was used in (a-g).


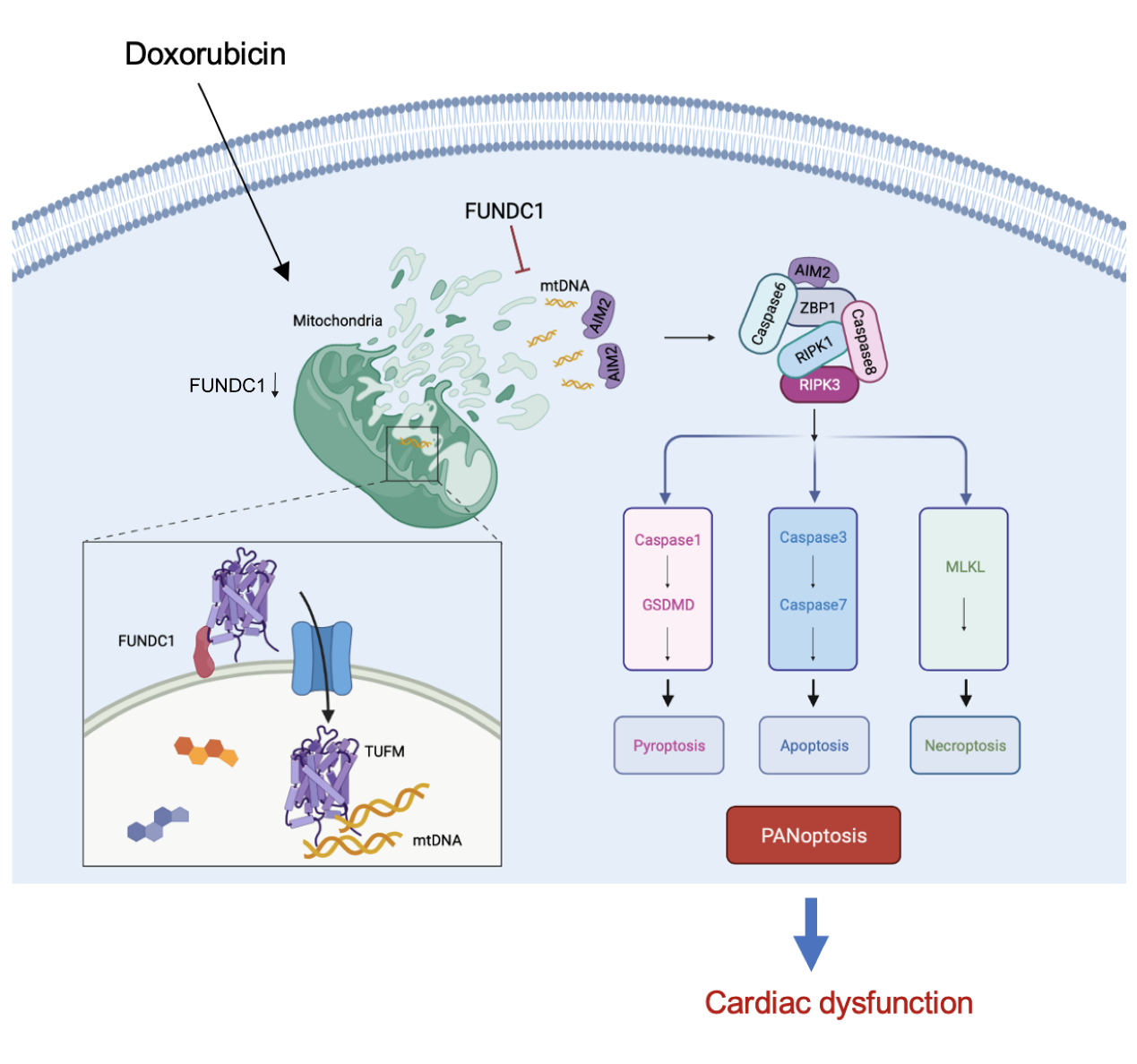


**Supplemental Fig.S7** Schematic diagram depicting proposed mechanism behind FUNDC1-evoked responses in DOX cardiotoxicity. FUNDC1 restrains mtDNA release to cytoplasm by direct binding with and recruiting TUFM to mitochondria to defend against mtDNA damage and cytosolic release in the face of DOX insult, resulting in deactivation PANoptosome and PANoptosis, and protection against cardiac dysfunction induced by DOX.
