# Supplementary figures and images for: FUNDC1 protects against doxorubicin-induced cardiomyocyte PANoptosis through stabilizing mtDNA via interaction with TUFM
Source: Cell Death Dis. 2022 Dec 5;13(12):1020. doi: 10.1038/s41419-022-05460-x (PMC9723119; doi:10.1038/s41419-022-05460-x)

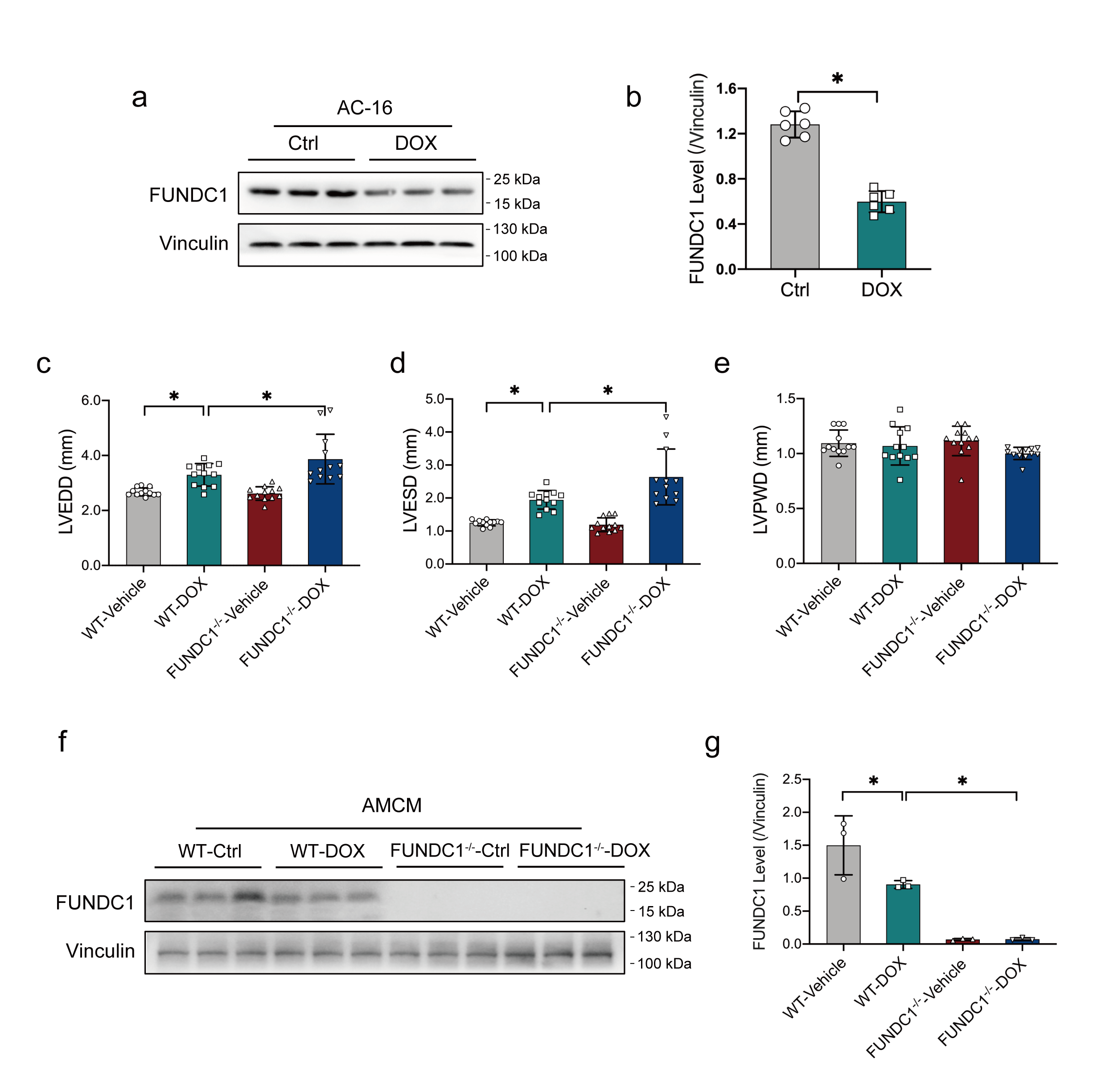

Supplement: Supplementary file 2 — Figure s1 [file 41419_2022_5460_MOESM2_ESM.tif]

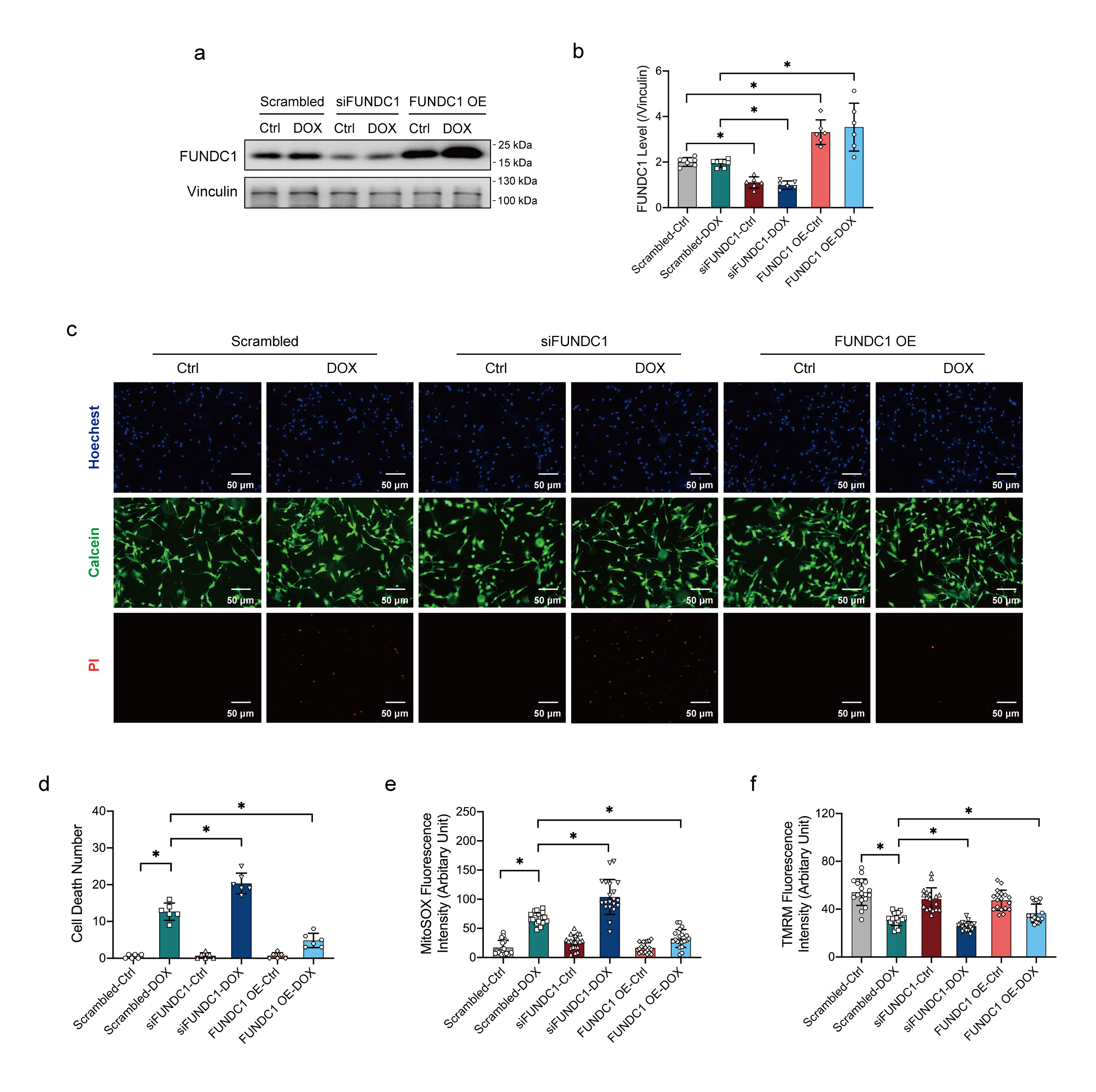

Supplement: Supplementary file 3 — Figure s2 [file 41419_2022_5460_MOESM3_ESM.tif]

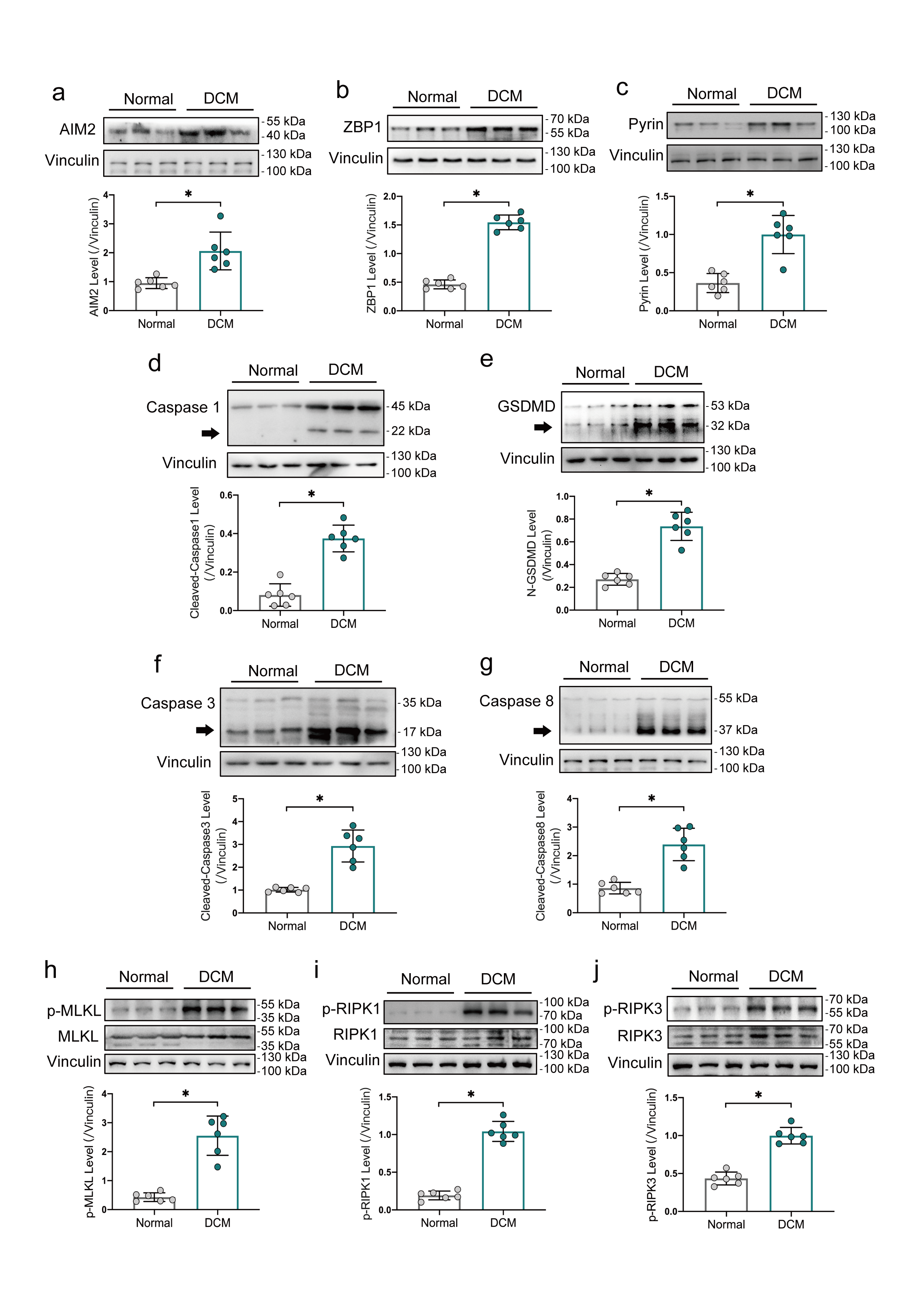

Supplement: Supplementary file 4 — Figure s3 [file 41419_2022_5460_MOESM4_ESM.tif]

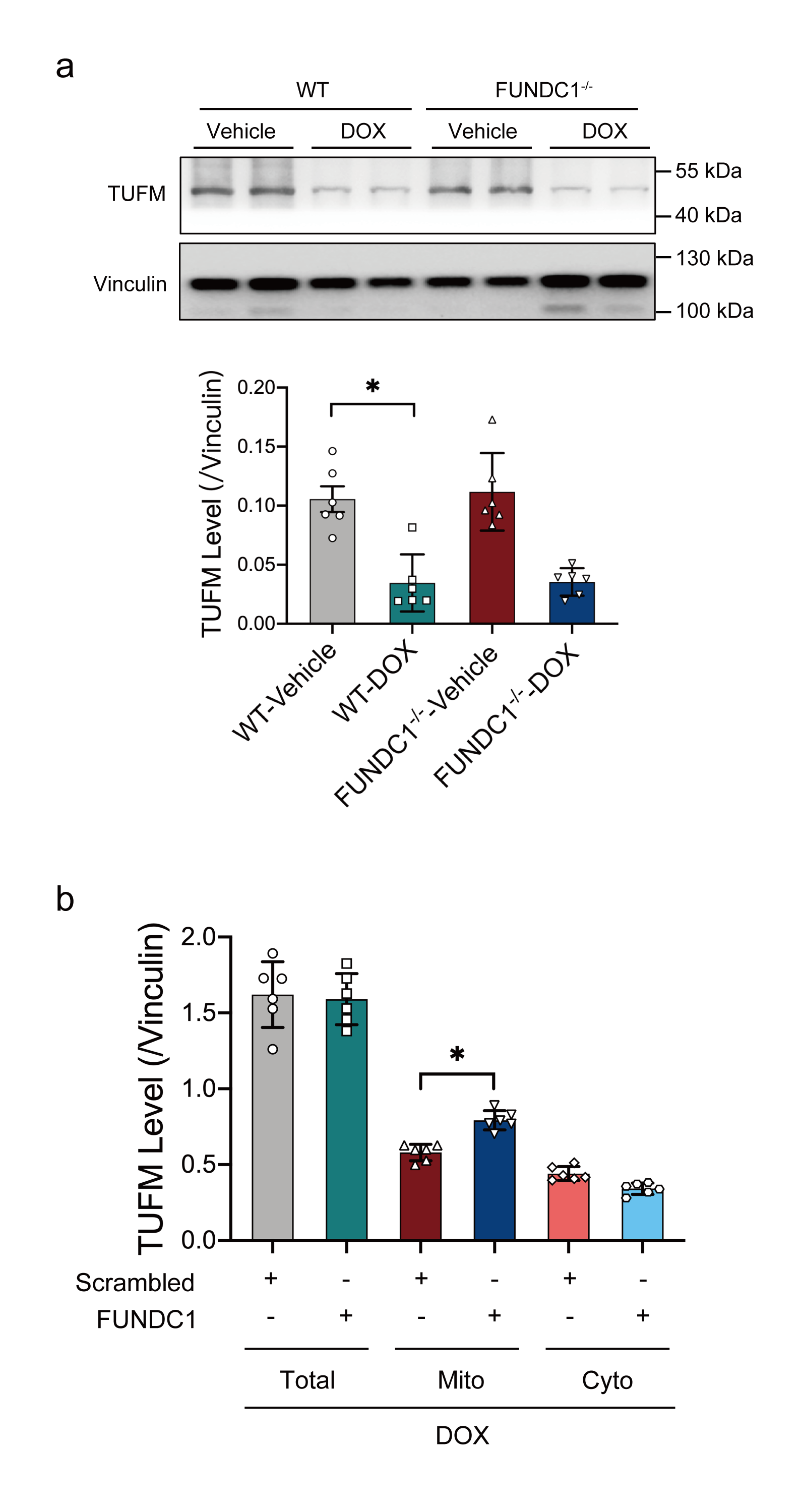

Supplement: Supplementary file 5 — Figure s4 [file 41419_2022_5460_MOESM5_ESM.tif]

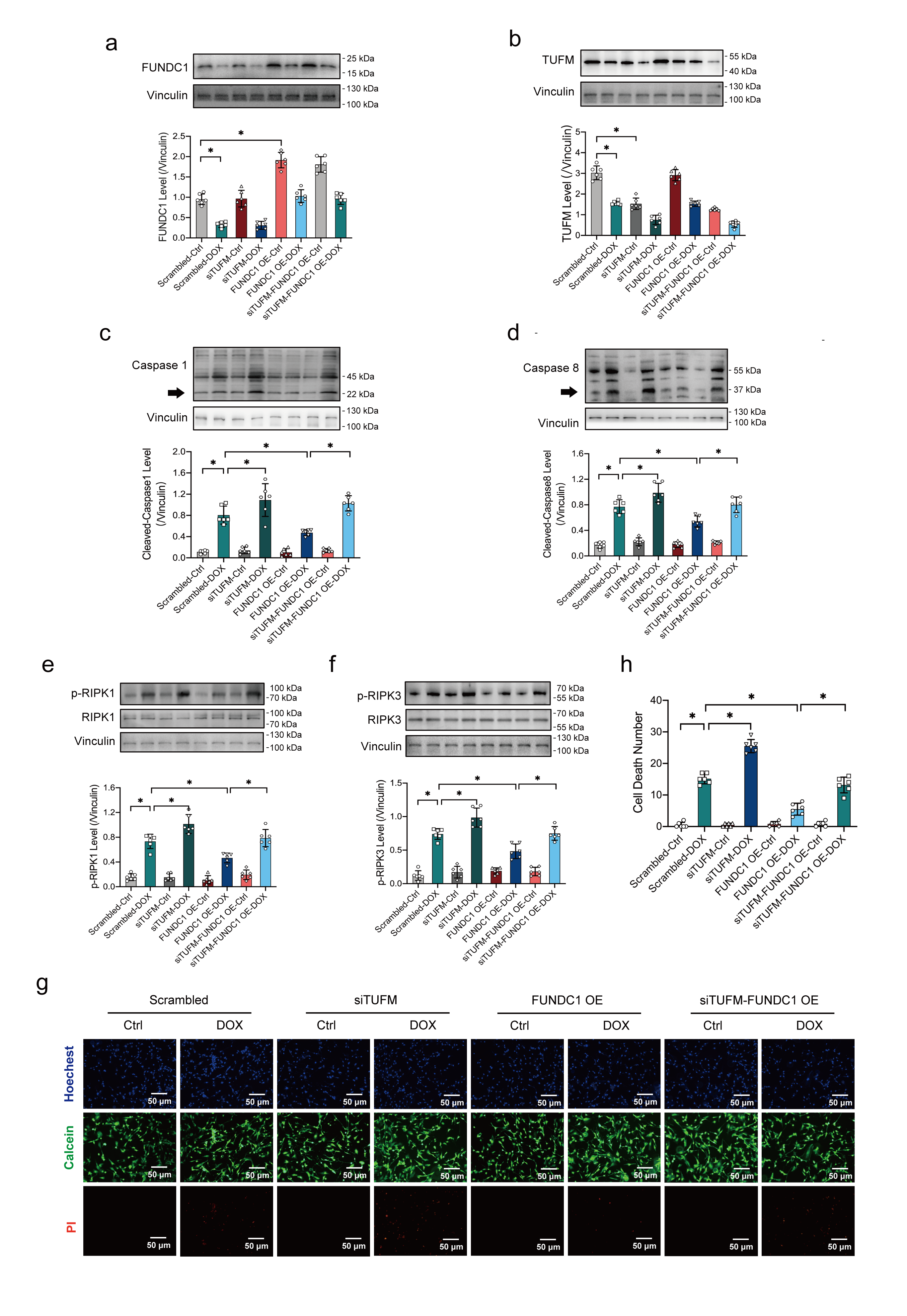

Supplement: Supplementary file 6 — Figure s5 [file 41419_2022_5460_MOESM6_ESM.tif]

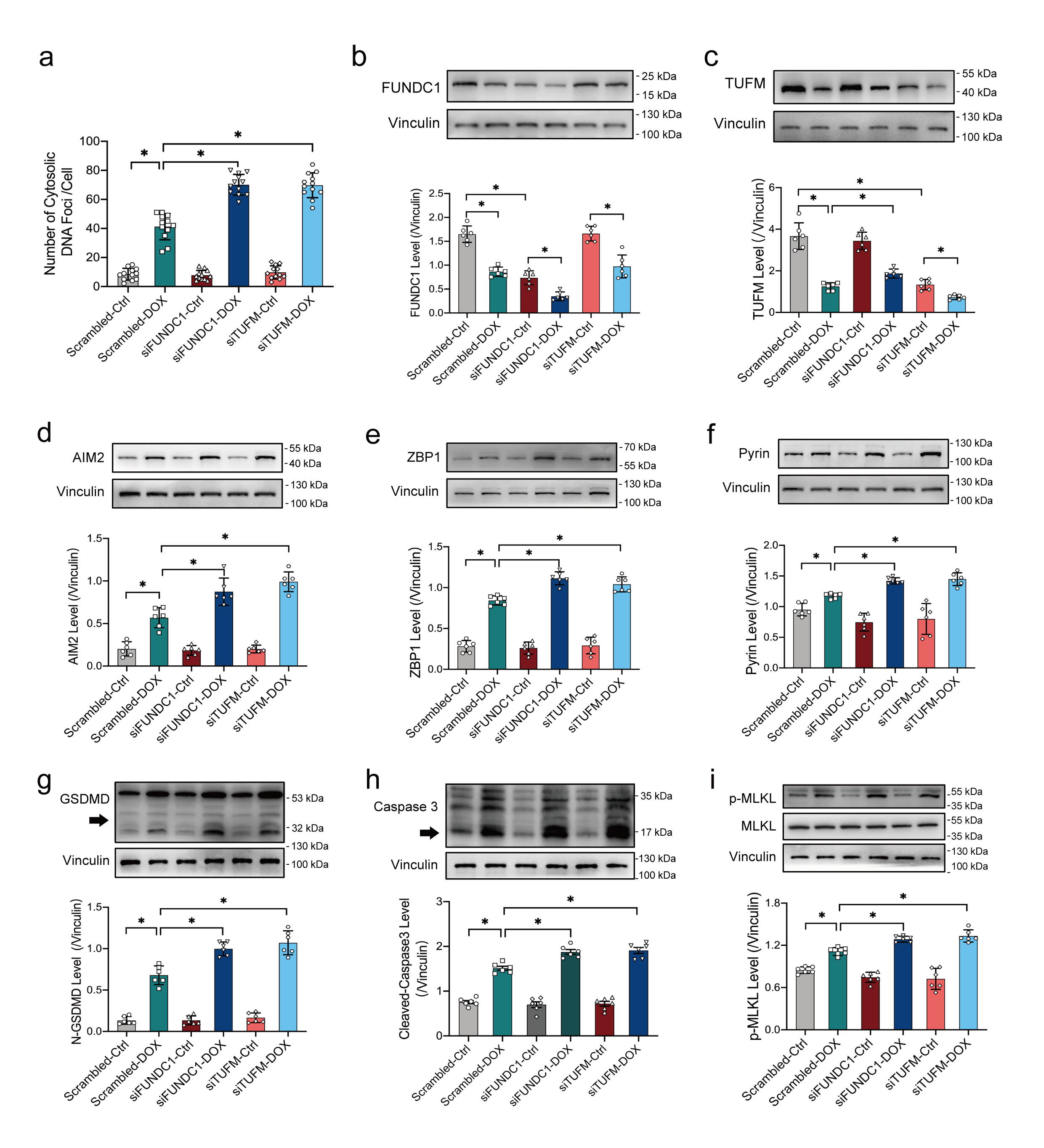

Supplement: Supplementary file 7 — Figure s6 [file 41419_2022_5460_MOESM7_ESM.tif]

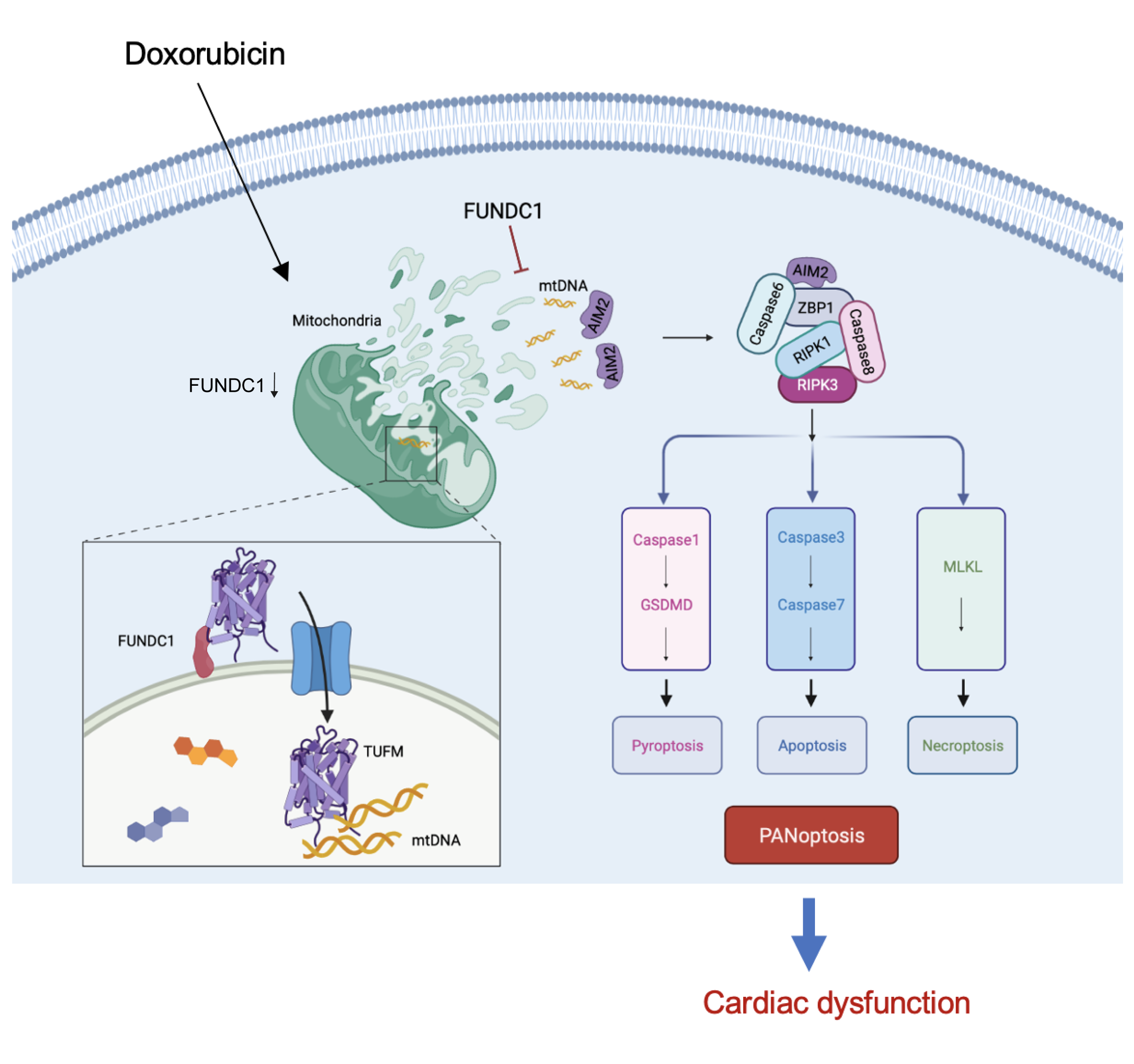

Supplement: Supplementary file 8 — Figure s7 [file 41419_2022_5460_MOESM8_ESM.tif]
