# Supplementary material for: FUNDC1 protects against doxorubicin-induced cardiomyocyte PANoptosis through stabilizing mtDNA via interaction with TUFM
Source: Cell Death Dis. 2022 Dec 5;13(12):1020. doi: 10.1038/s41419-022-05460-x (PMC9723119; doi:10.1038/s41419-022-05460-x)

# Fig.1

Fig.1a

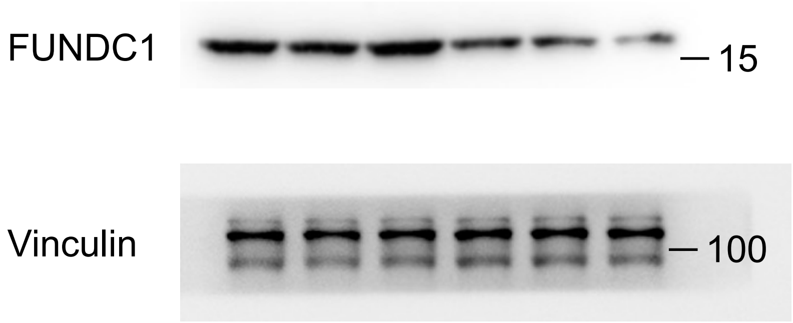


Fig.1d

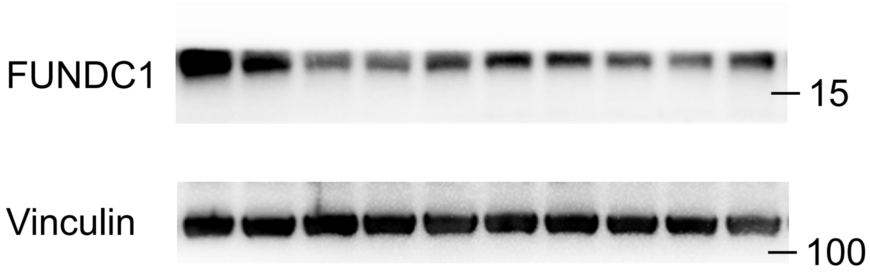


Fig.1g

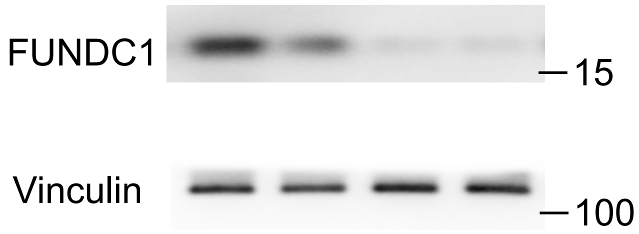


# Fig.5

Fig.5b

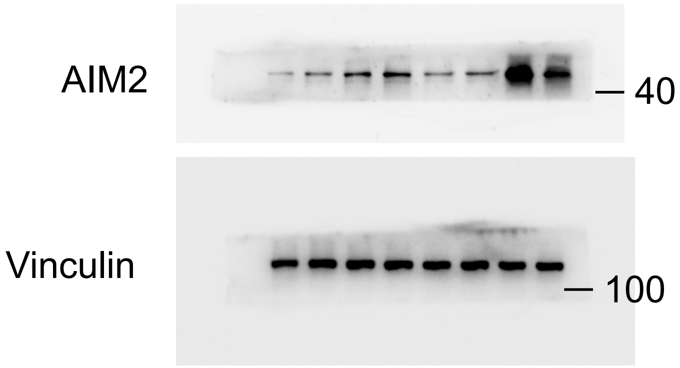


Fig.5c

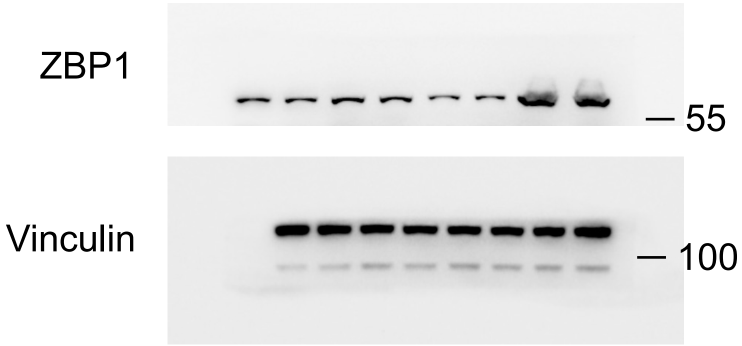


Fig.5d

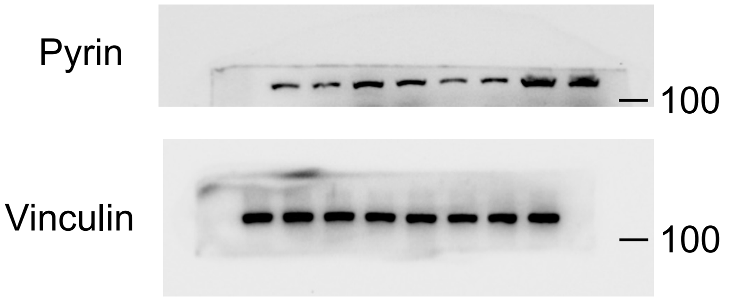

**Fig.5**

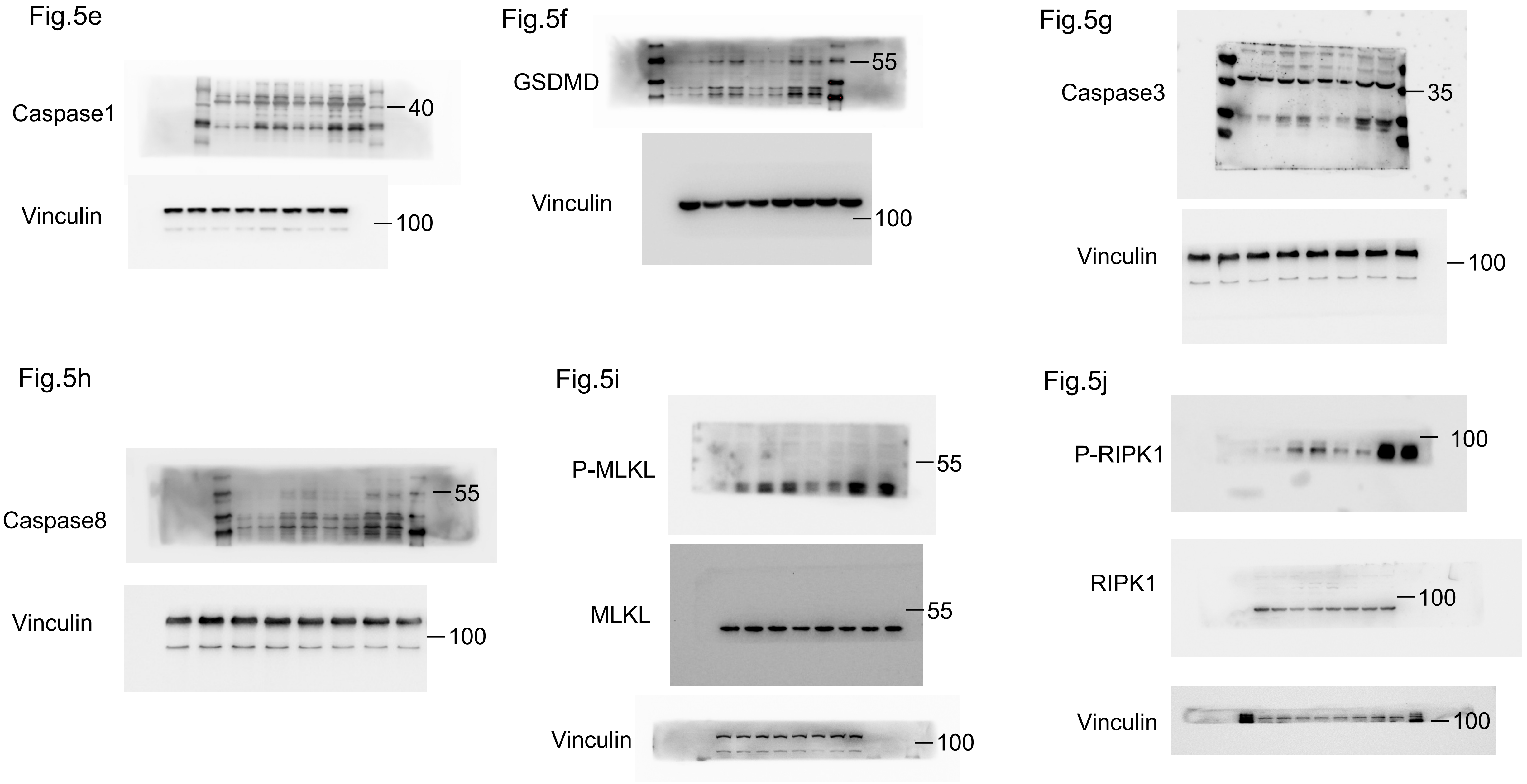

Fig.5e
Caspase1
40
Vinculin
100
Fig.5f
GSDMD
55
Vinculin
100
Fig.5g
Caspase3
35
Vinculin
100
Fig.5h
Caspase8
55
Vinculin
100
Fig.5i
P-MLKL
55
MLKL
55
Vinculin
100
Fig.5j
P-RIPK1
100
RIPK1
100
Vinculin
100

Fig.5k

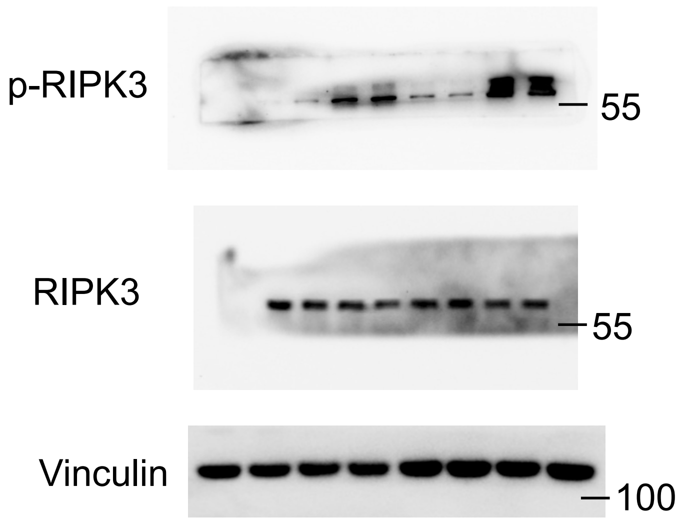


Fig.5l

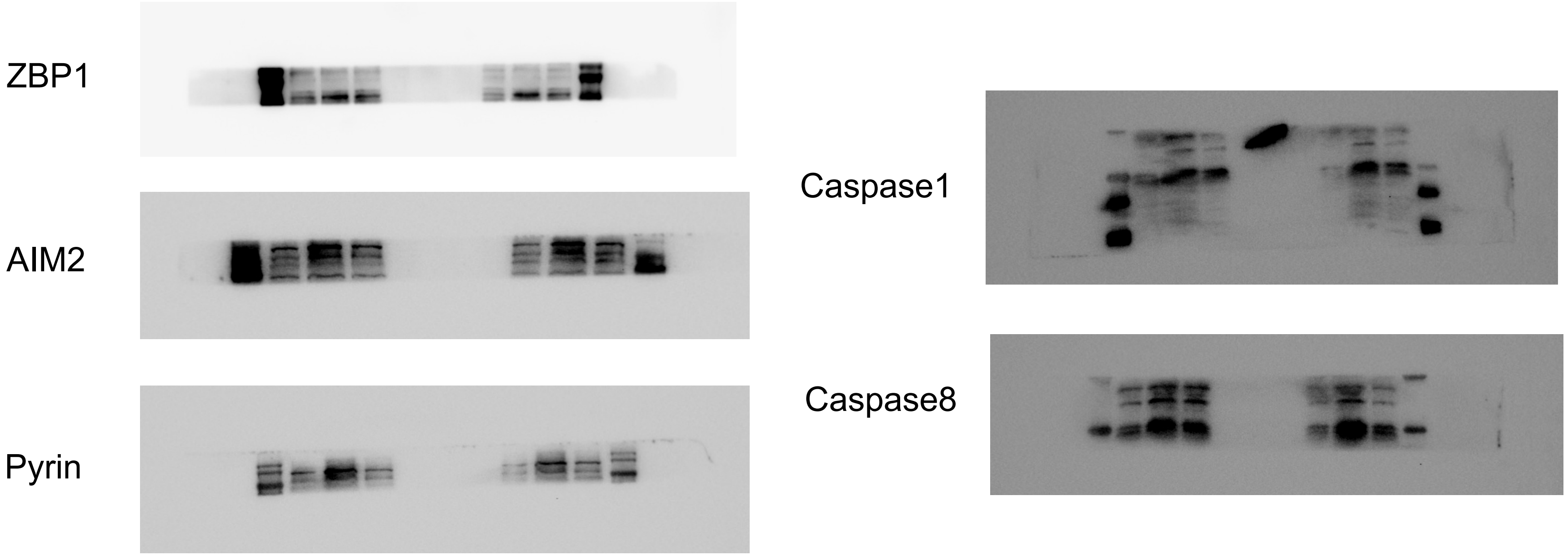


Fig.5l

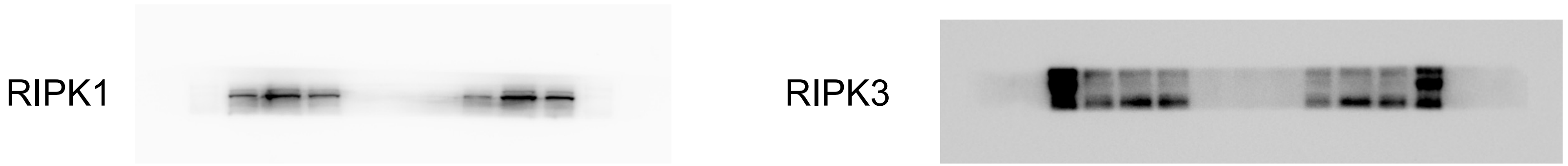

Fig.5m

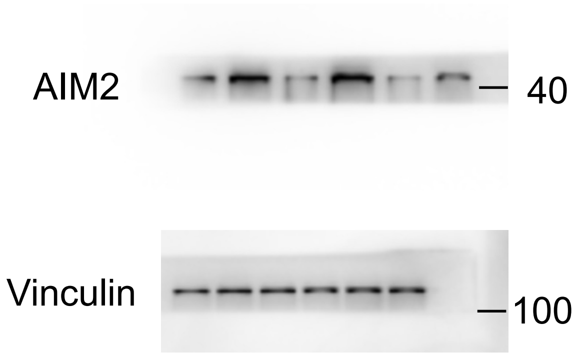


Fig.5n

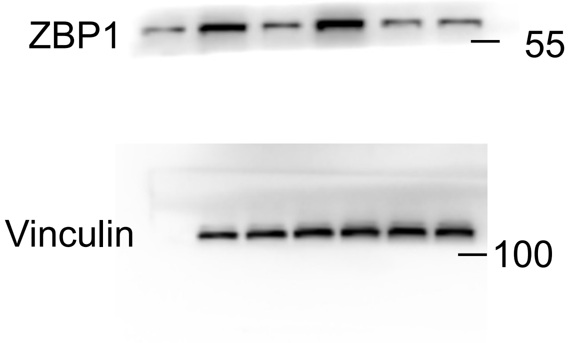


Fig.5o

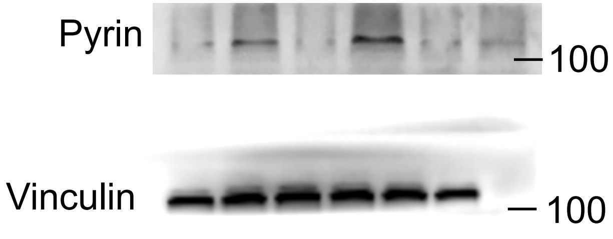


# Fig.6

Fig.6a

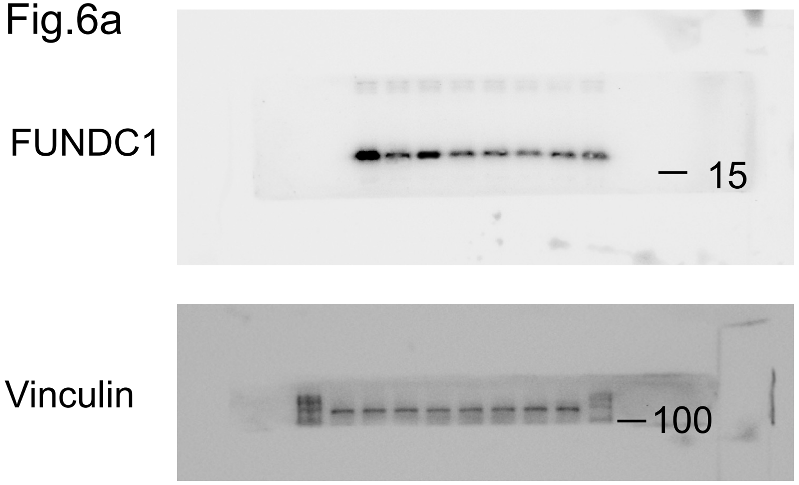


Fig.6c

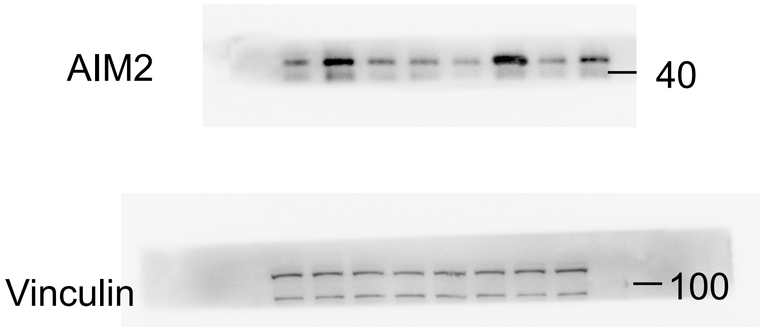


Fig.6d

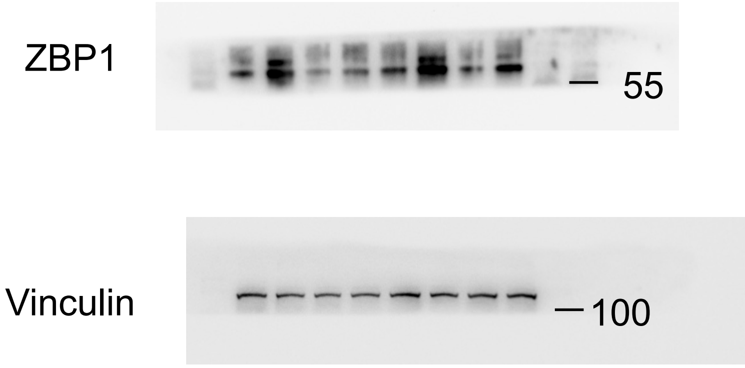

Fig.6e

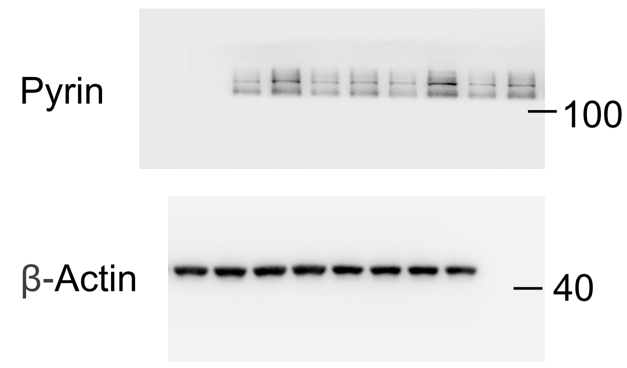


Fig.6f

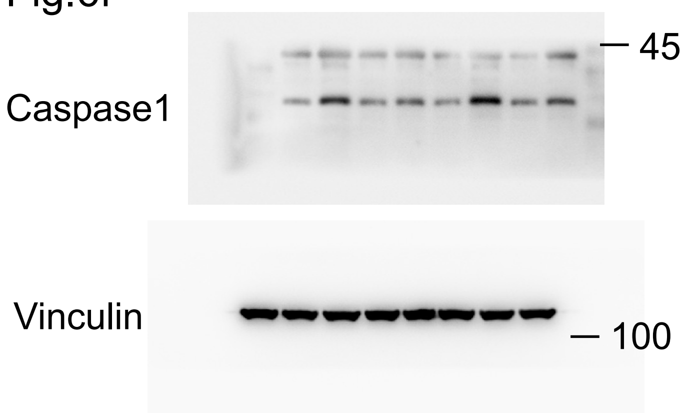


Fig.6g

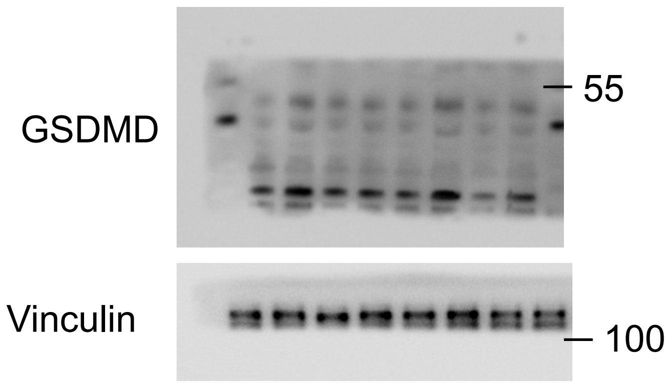


Fig.6h

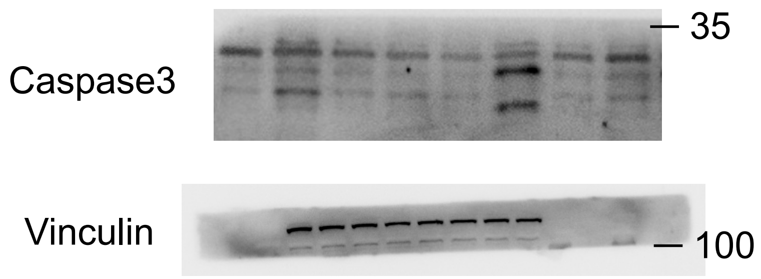


Fig.6i

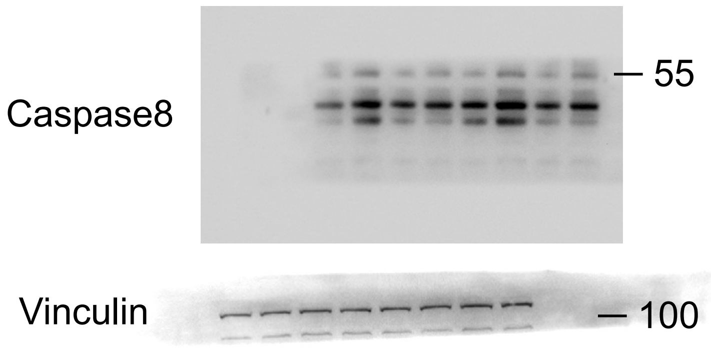


Fig.6j

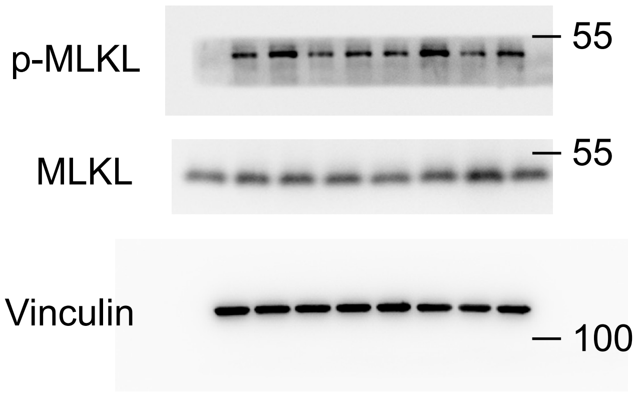

Fig.6k

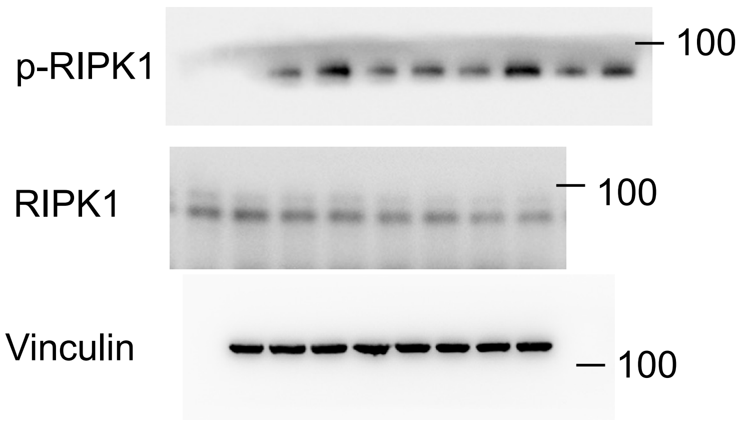


Fig.6l

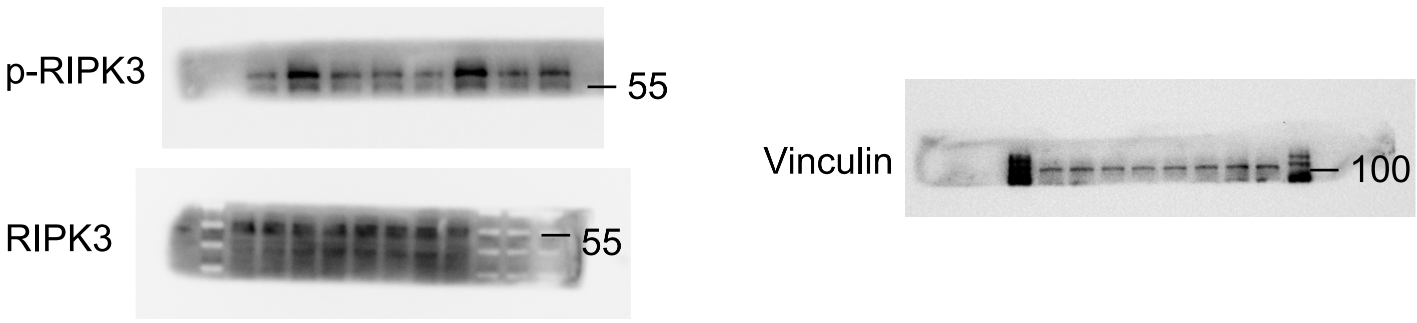


## Fig.7

Fig.7j

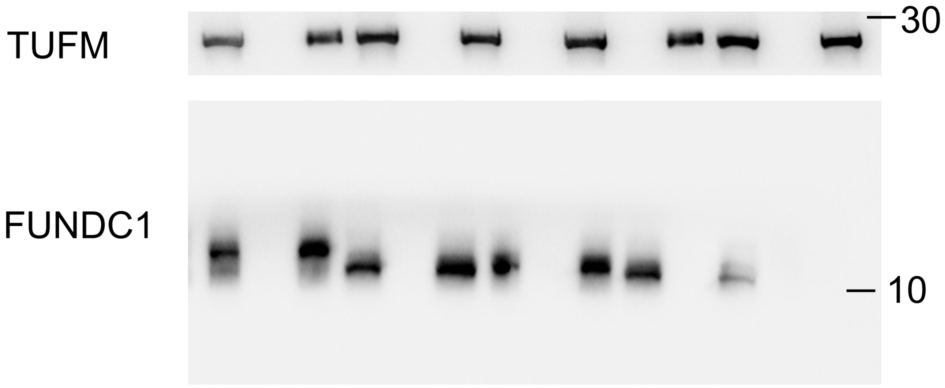


Fig.7k

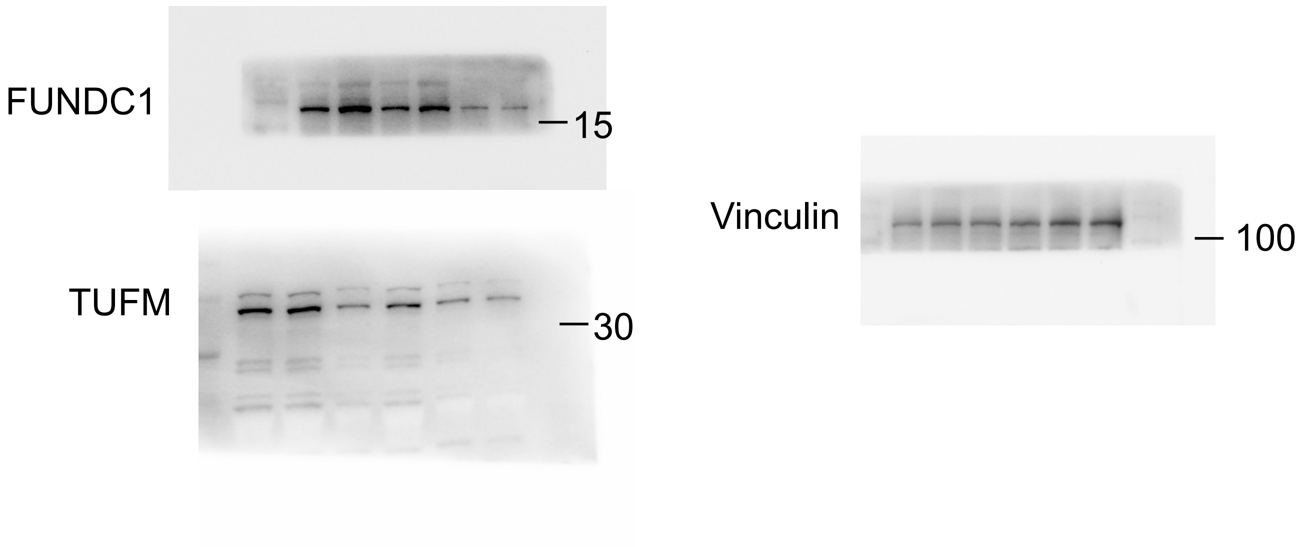

**Fig.8**

Fig.8d

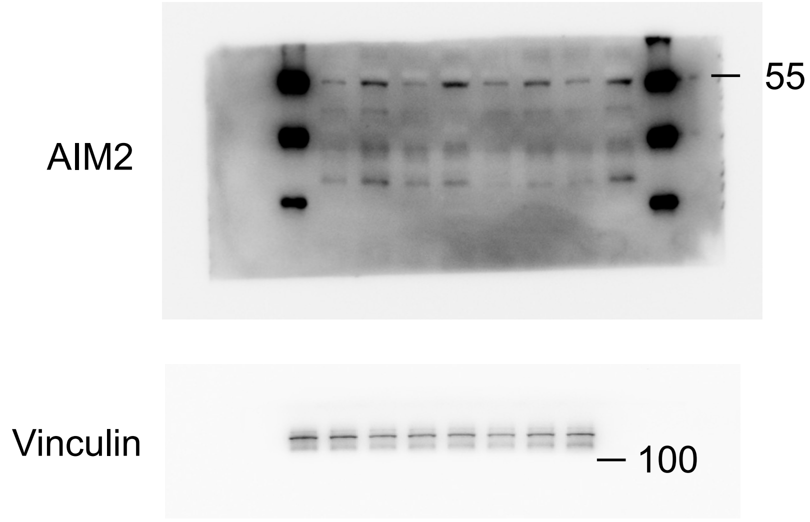


Fig.8e

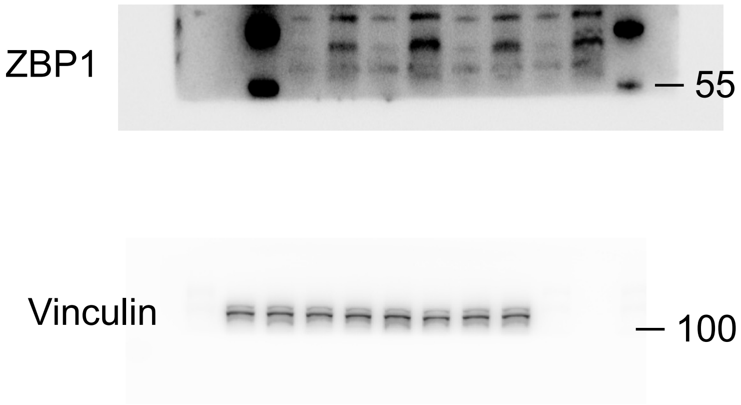


Fig.8f

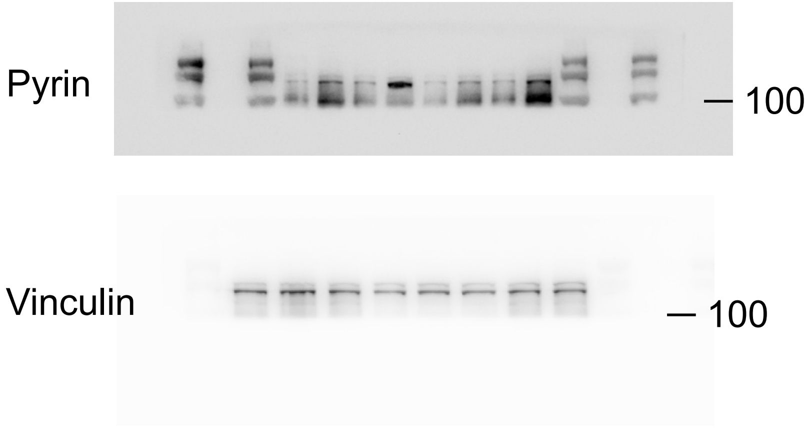


Fig.8g

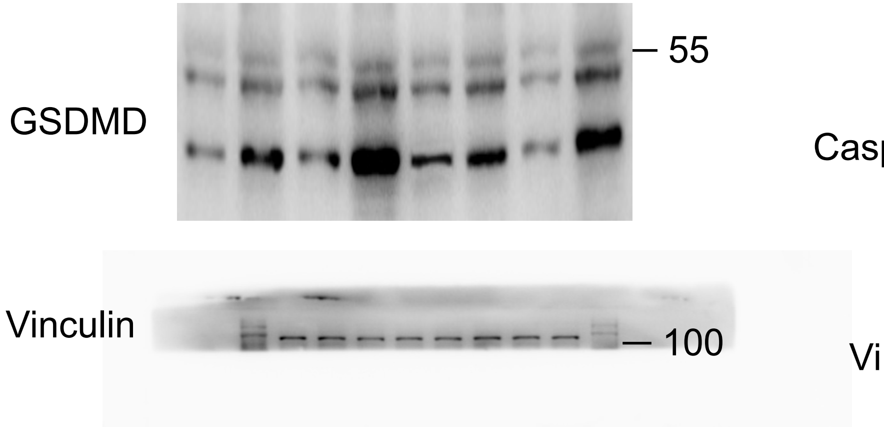


Fig.8h

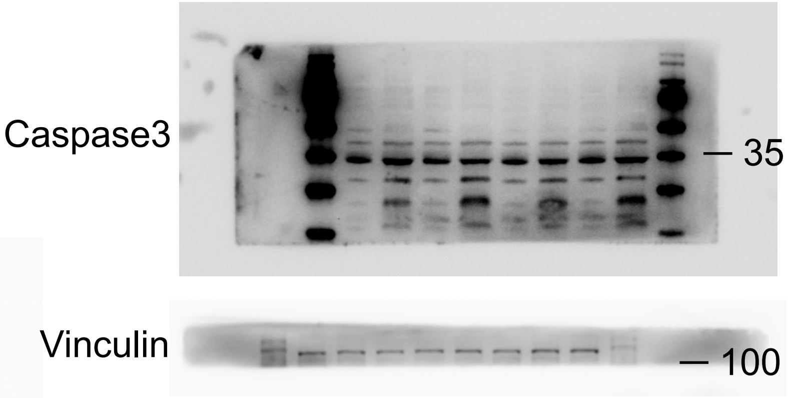


Fig.8i

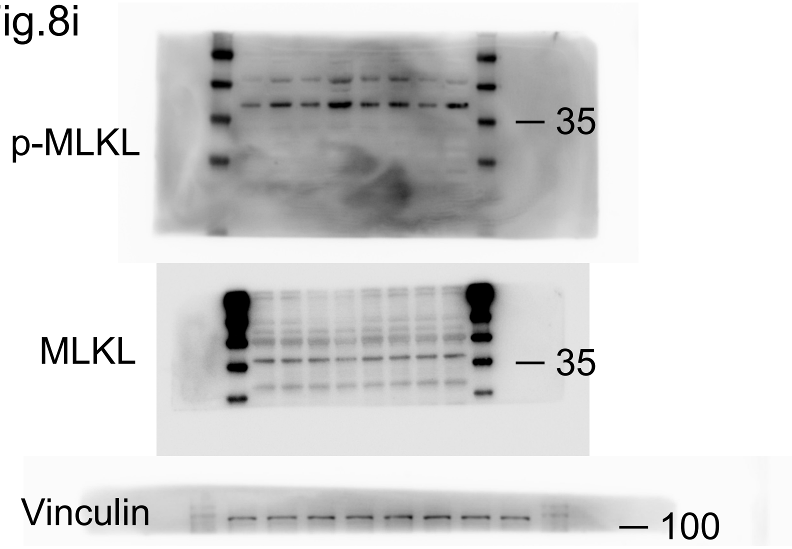

## Fig.S1

Fig.S1a

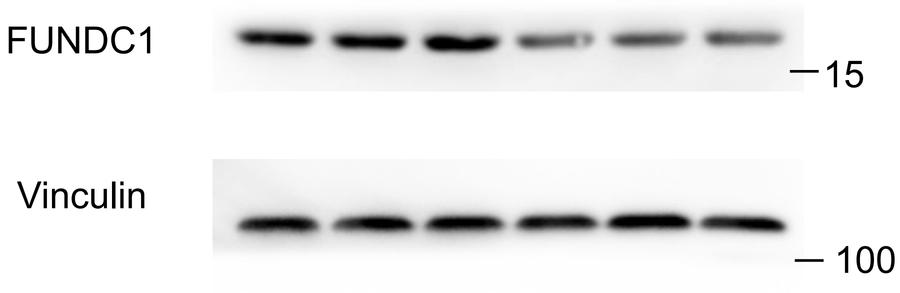


Fig.S1b

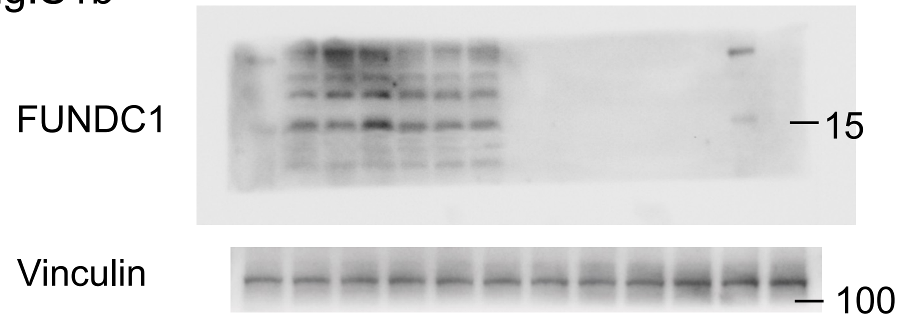


## Fig.S2

Fig.S2a

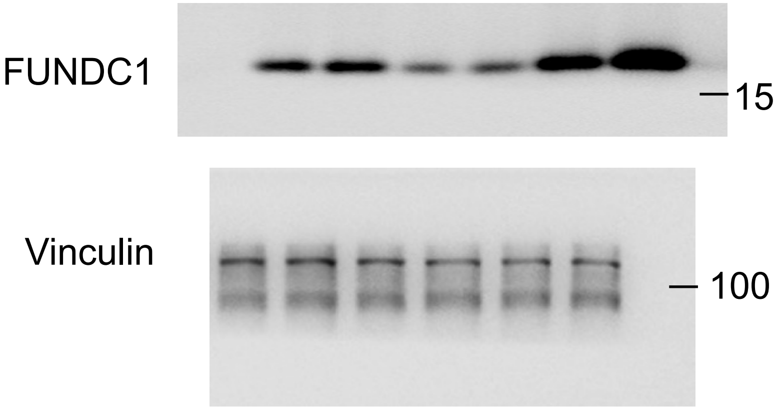

**Fig.S3**

Fig.S3a

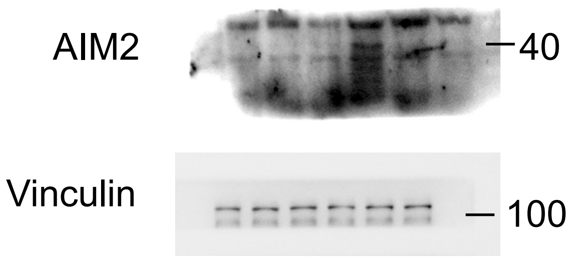


Fig.S3b

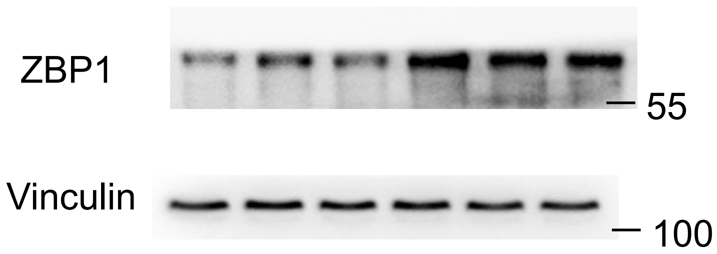


Fig.S3c

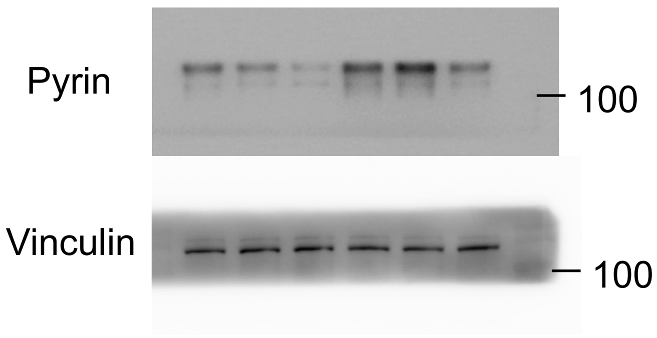


Fig.S3d

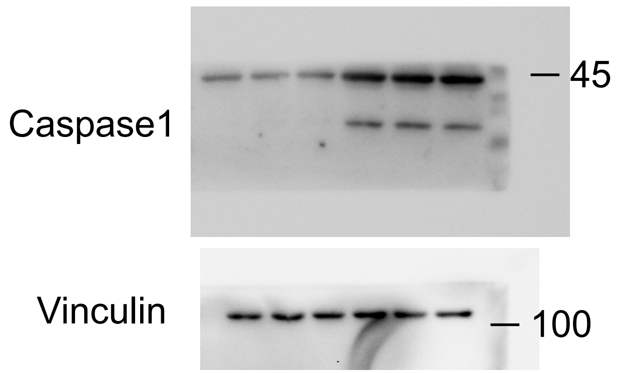


Fig.S3e

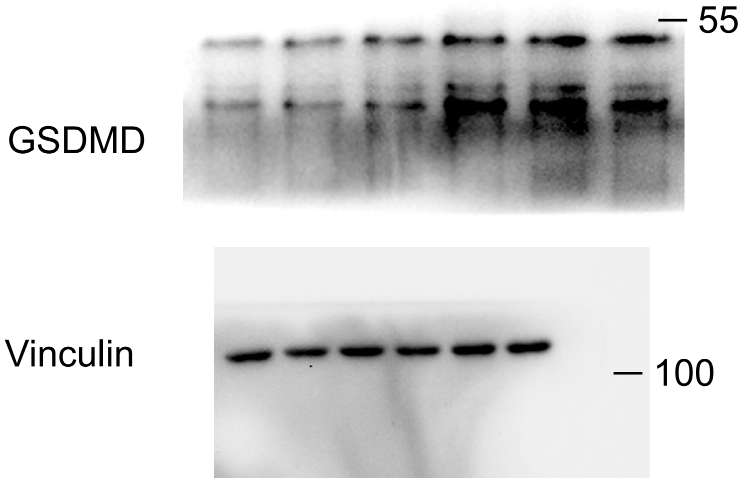


Fig.S3f

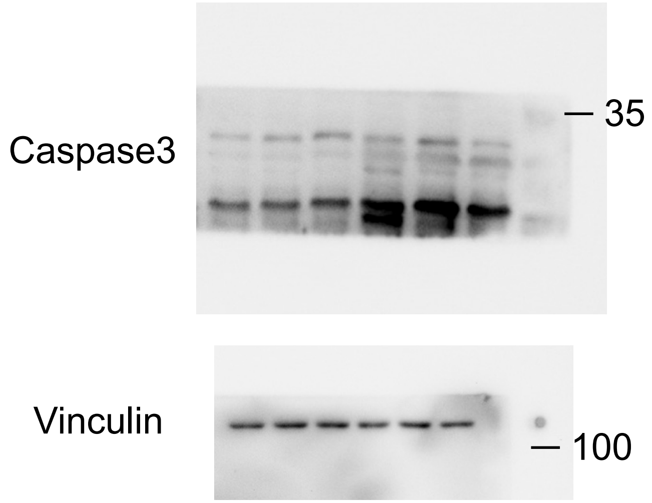

Fig.S3g

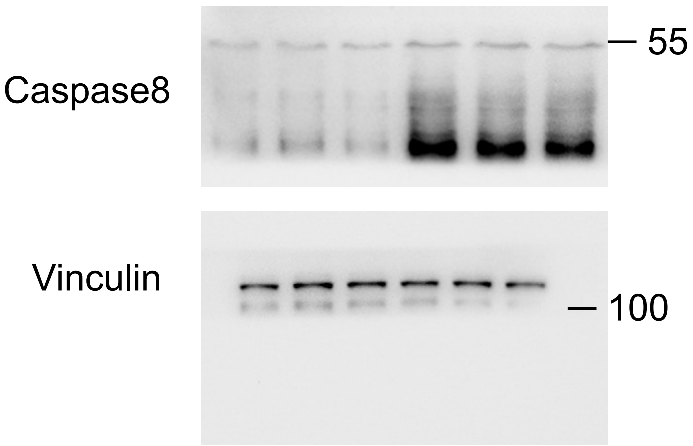


Fig.S3h

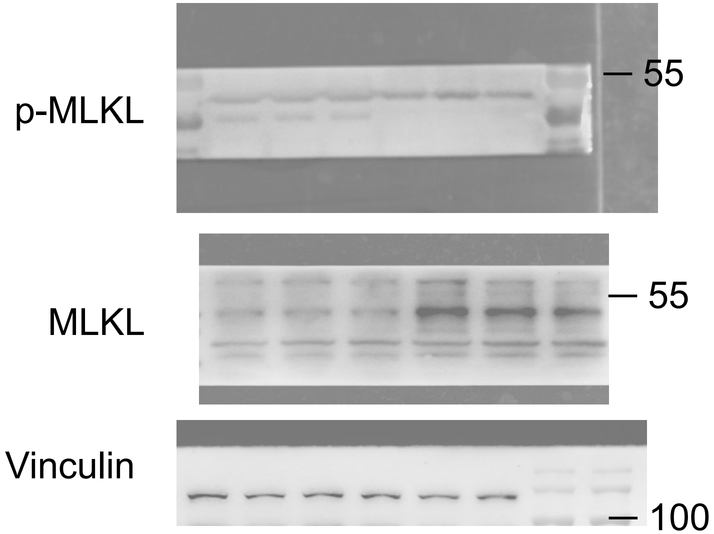


Fig.S3i

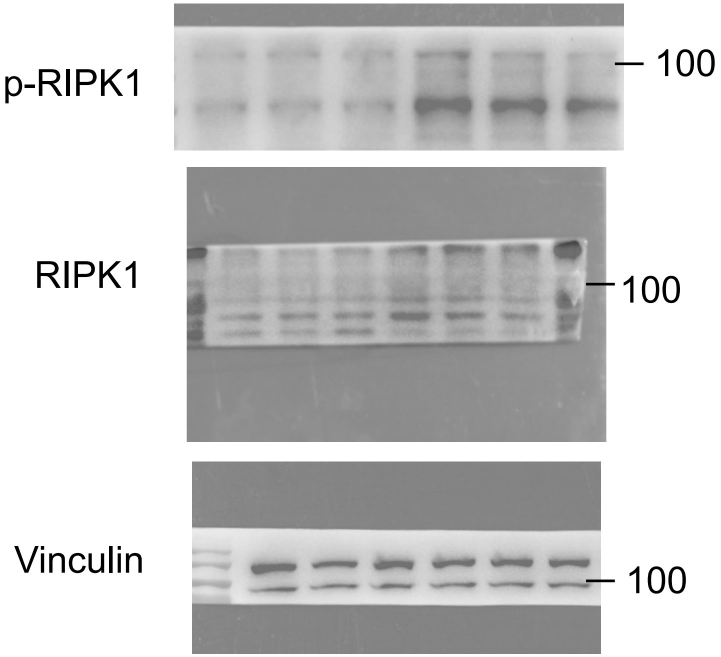


Fig.S3j

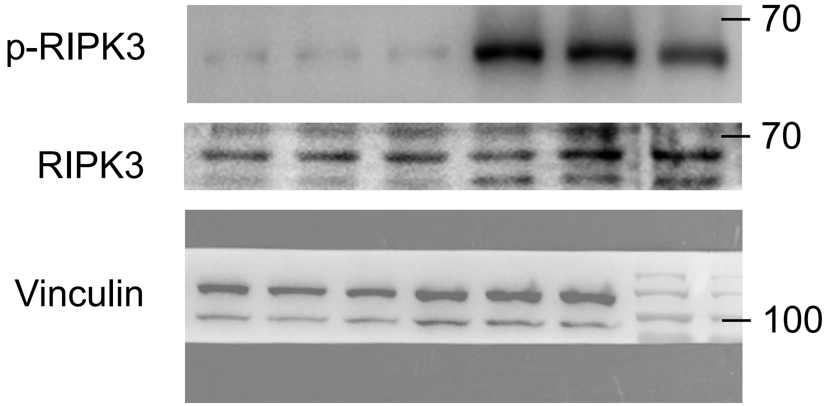


**Fig.S4**

Fig.S4a

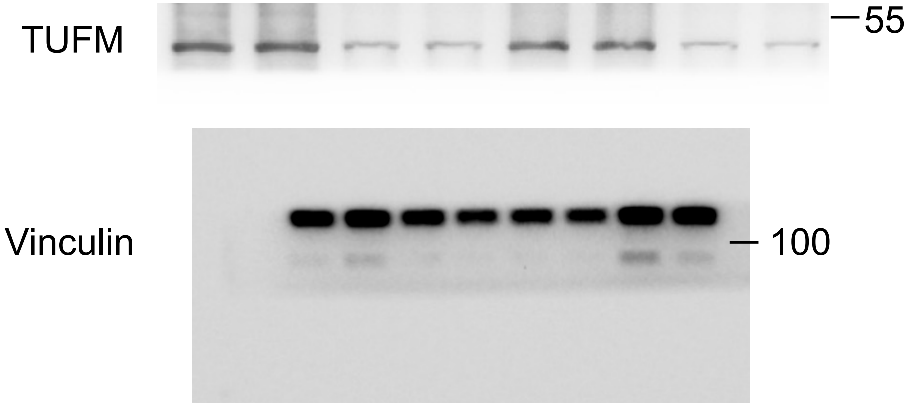

**Fig.S5**

Fig.S5a

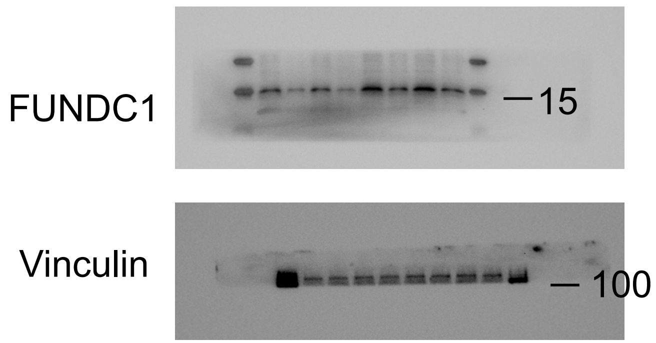


Fig.S5b

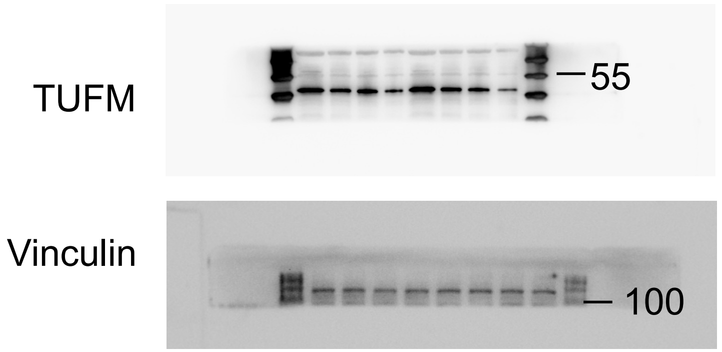


Fig.S5c

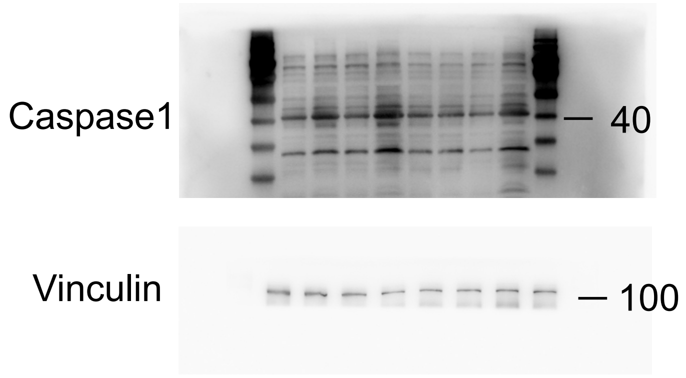


Fig.S5d

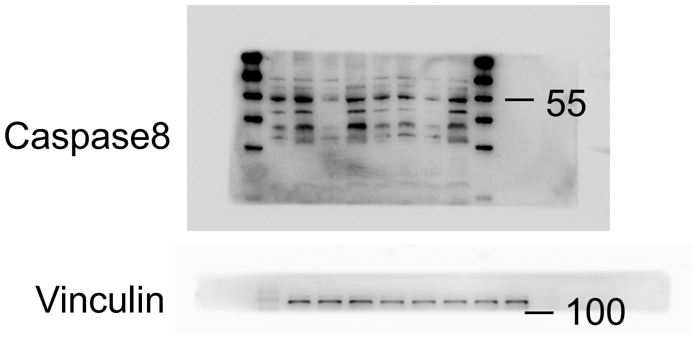


Fig.S5e

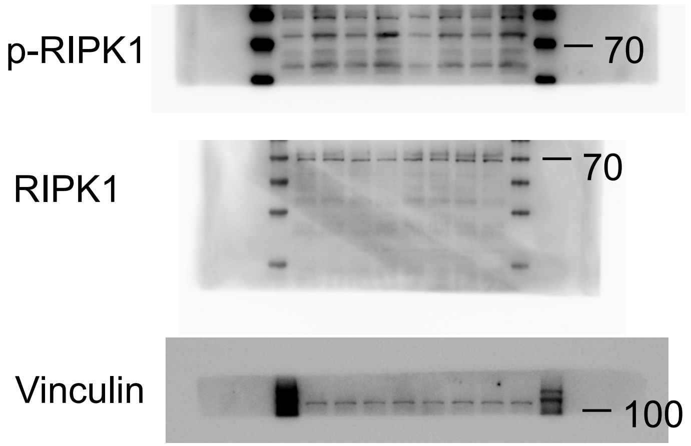


Fig.S5f

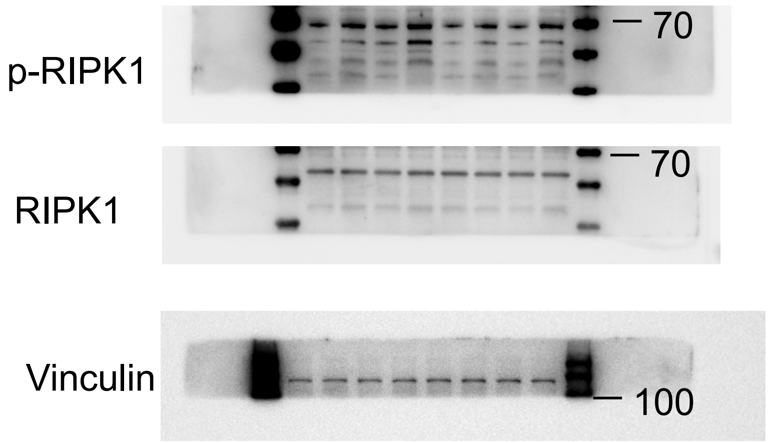

**Fig.S6**

Fig.S6b

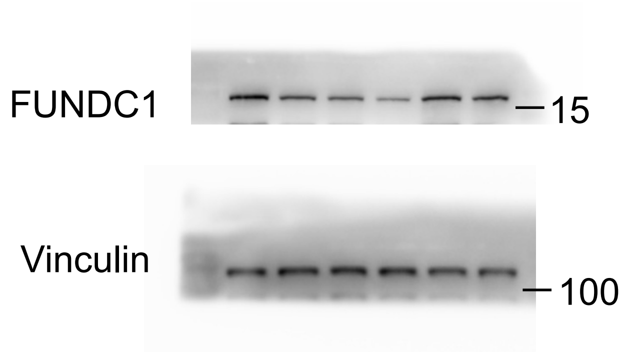


Fig.S6c

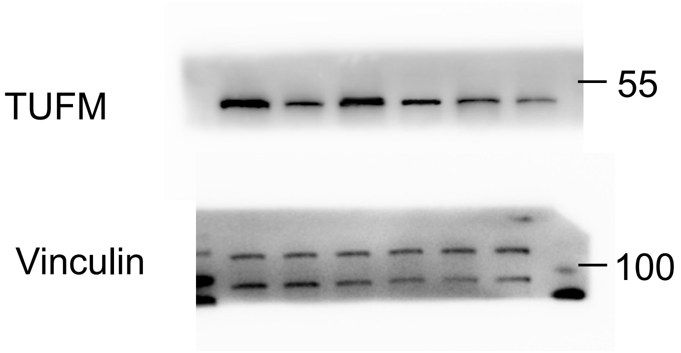


Fig.S6d

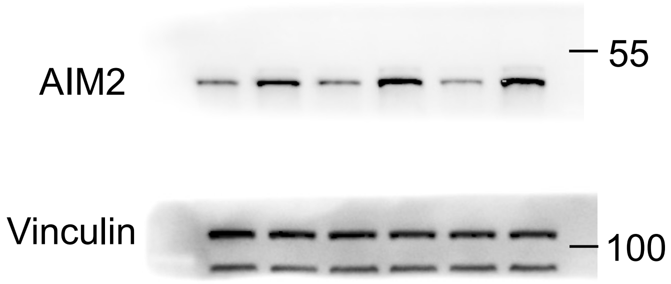


Fig.S6e

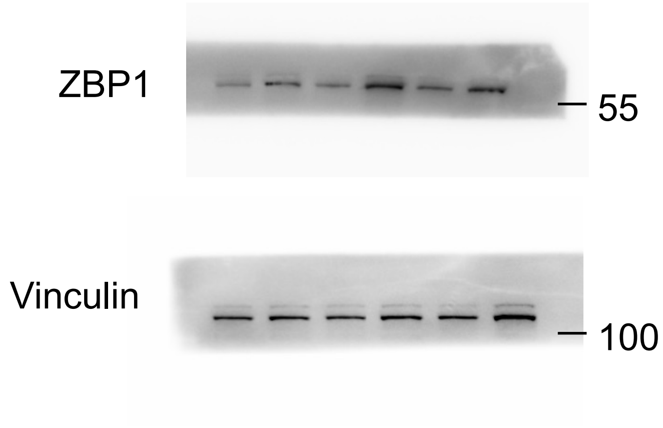


Fig.S6f

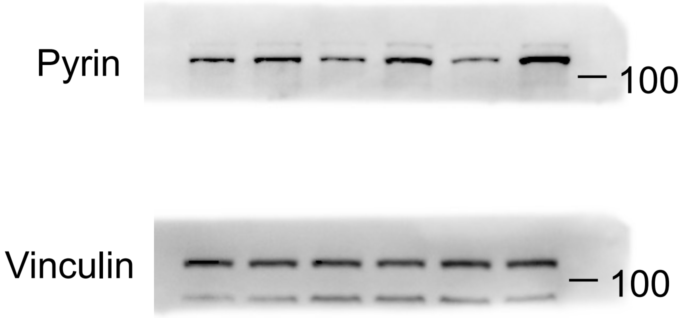


Fig.S6g

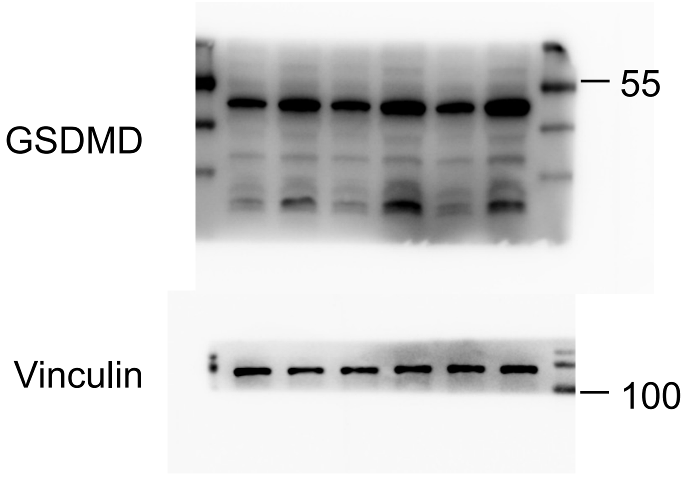

Fig.S6h

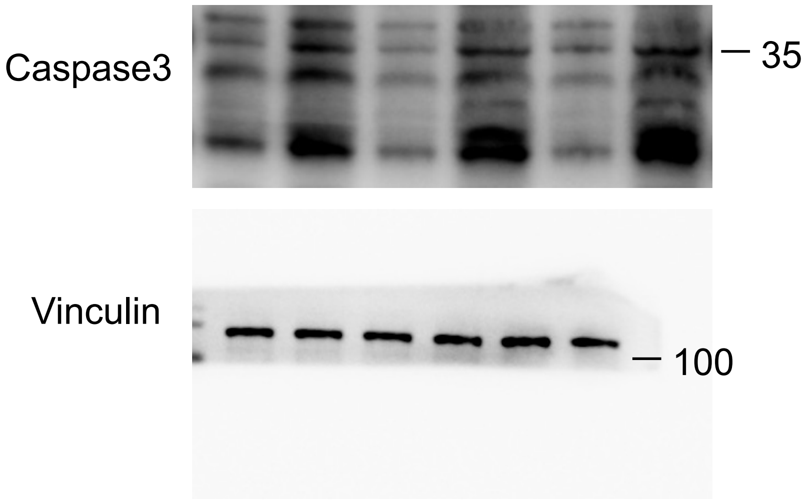


Fig.S6i

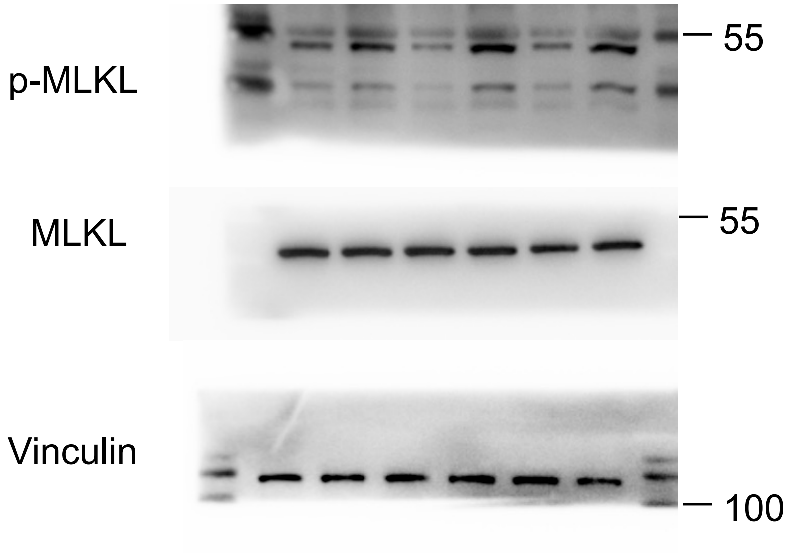

Supplement: Supplementary file 9 — western blots [file 41419_2022_5460_MOESM9_ESM.pdf]
